# From Pre-Implementation to Institutionalization: Lessons From Sustaining a Perinatal Audit Program in South Africa

**DOI:** 10.9745/GHSP-D-22-00213

**Published:** 2023-04-28

**Authors:** Mary V. Kinney, Asha S. George, Natasha R. Rhoda, Robert C. Pattinson, Anne-Marie Bergh

**Affiliations:** a School of Public Health, University of the Western Cape, Bellville, South Africa.; b Mowbray Maternity Hospital, Faculty of Health Sciences, University of Cape Town, Cape Town, South Africa.; c Maternal and Infant Health Care Strategies Research Unit, South African Medical Research Council and Faculty of Health Sciences, University of Pretoria, Pretoria, South Africa.

## Abstract

**Introduction:**

Maternal and perinatal death surveillance and response (MPDSR), or related forms of maternal and perinatal death audits, can strengthen health systems. We explore the history of initiating, scaling up, and institutionalizing a national perinatal audit program in South Africa.

**Methods:**

Data collection involved 56 individual interviews, a systematic document review, administration of a semistructured questionnaire, and 10 nonparticipant observations of meetings related to the perinatal audit program. Fieldwork and data collection in the subdistricts occurred from September 2019 to March 2020. Data analysis included thematic content analysis and application of a tool to measure subdistrict-level implementation. This study expands on case study research applied to 5 Western Cape subdistricts with long histories of implementation.

**Results:**

Although established in the early 1990s, the perinatal audit program was not integrated into national policy and guidelines until 2012 but was then excluded from policy in 2021. A network of national and subnational structures that benefited from a continuity of actors evolved and interacted to support uptake and implementation. Intentional efforts to demonstrate impact and enable local adaptation allowed for more ownership and buy-in. Implementation requires continuous efforts. Even in 5 subdistricts with long histories of practice, we found operational gaps, such as incomplete meeting minutes, signaling a need for strengthening. Nevertheless, the tool used to measure implementation may require revisions, particularly in settings with institutionalized practice.

**Conclusion:**

This article provides lessons on how to initiate, expand, and strengthen perinatal audit. Despite a long history of implementation, the perinatal audit program in South Africa cannot be assumed to be indefinitely sustainable or final in its current form. To monitor uptake and sustainability of MPDSR, including perinatal audit, we need research approaches that allow exploration of context, local adaptation, and underlying issues that support sustainability, such as relationships, leadership, and trust.

## Abstract (precis)

The authors explored the implementation history of South Africa’s perinatal audit program and identified lessons learned, policy and operational gaps, and issues with an existing measurement tool found while measuring the program’s implementation in 5 districts.

## Teaser

**Key Findings:**

- Lessons learned from implementing a perinatal audit program in South Africa showed the importance of embedding the process within the health system, multiple structures with a continuity of actors and government ownership, demonstration of impact, local adaptation, and continuous efforts to institutionalization.
- Using a tool to measure the program’s implementation in 5 subdistricts in the Western Cape Province identified operational gaps in the perinatal audit program as well as possible gaps within the tool itself.

**Key Implications:**

- Even in settings with wide uptake and use, such as South Africa, institutionalizing a national perinatal audit program requires continuous efforts to build the network of actors and governance structures, generate buy-in, and integrate into policy and guidelines.
- Measuring implementation of perinatal audit at the facility level using the existing tool is not sufficient for understanding sustainable and quality practice.

## BACKGROUND

Achieving the Sustainable Development Goal for health requires increased attention on high-quality health systems.^1^ Perinatal mortality (fetal death of at least 28 weeks of gestation and/or 1,000 g in weight and newborn deaths up to 7 days after birth) can serve as a sensitive marker of a health system’s inability to provide quality care and respond to care delays.^2^^–^^4^ With growing attention to perinatal mortality,^5^ the World Health Organization and partners have integrated perinatal audit with maternal death surveillance and response to streamline efforts and strengthen health systems.^6^ Now referred to as “maternal and perinatal death surveillance and response” (MPDSR), this intervention process aims to continuously ensure deaths are notified and data around each death are collected, analyzed, and reviewed to investigate the cause and circumstances surrounding each death in order to identify actions that may strengthen the health system and prevent future deaths.^7^ There is a need to better understand implementation practice, scale, and impact of MPDSR, including the perinatal component, as more low- and middle-income countries (LMICs) adapt national policies and strengthen implementation efforts.^8^^–^^12^

There is a need to better understand implementation practice, scale, and impact of MPDSR as more LMICs adapt national policies and strengthen implementation efforts.

A scoping review on MPDSR implementation identified 58 studies from LMICs.^13^ Most of these studies focused on maternal death review or maternal death surveillance and response; 12 studies were on combined maternal and perinatal audits; and 5 studies focused only on perinatal audit. Implementation factors were similar for both types of audit and included service delivery factors (tangible inputs), such as trainings and focal persons, as well as societal and systems factors, such as networks, team dynamics, and individual motivation.^13^^,^^14^ Implementation occurs at all levels of the health system—national, subnational, facility, and community^15^—and is a complex social process influenced by a multitude of factors.^16^^–^^18^ The scoping review revealed gaps in the literature, particularly around how the implementation process works and why, and found that few countries report robust operational systems at scale, especially for perinatal audit.^6^^,^^13^^,^^19^^,^^20^ South Africa is 1 exception where perinatal audits have been implemented for a long time with some success.^21^^–^^23^

As other LMICs seek to introduce, scale up, and strengthen perinatal audit as part of MPDSR, it may be helpful to learn from this exemplar. With this article, we aim to provide a comprehensive assessment of South Africa’s experience of scaling and institutionalizing a perinatal audit program known as the Perinatal Problem Identification Program (PPIP) at national and subnational levels. We apply different theoretical approaches used in implementation research to describe, understand, and explain the sustainability of this program.^24^ We draw from original research based on the first author’s doctoral research,^13^^,^^25^^,^^26^ as well as build from other studies on implementation of the perinatal audit program in South Africa.^22^^,^^23^^,^^27^^,^^28^

## METHODS

South Africa is a middle-income country with just under 1 million births per year and a national perinatal mortality rate of 30.9 deaths per 1,000 total deliveries.^29^ Perinatal mortality rates, including newborn mortality and stillbirth rates, have experienced stagnation in the past decade after a reduction from 1990 to 2012.^29^^–^^31^ The Western Cape, the southwestern-most province in South Africa, has been using PPIP as a perinatal audit tool since 2000.^22^ With a long history of practice, the Western Cape and its facilities are a conducive environment to understand mechanisms influencing sustained implementation practice. The Western Cape had approximately 102,000 births and a perinatal mortality rate of 24.8 in 2019 according to routine data.^29^

The Western Cape includes 1 metropolitan municipality (Cape Town), which has a unique health system, and 5 districts, which are further subdivided into 24 local municipalities called subdistricts.^32^^,^^33^ District hospitals manage all of the deliveries in these subdistricts unless a referral is required. Outpatient services, such as antenatal and postnatal care, take place at primary health care facilities.^33^ The PPIP reporting structure in the Western Cape comprises 5 PPIP regions, which align to the 5 regional hospitals. Each PPIP region has a designated regional PPIP coordinator, often a specialist (e.g., obstetrician or pediatrician), who oversees implementation of perinatal audit in the subdistricts that they oversee as an outreach specialist (Supplement Figure 1). Selection criteria for subdistricts included: (1) currently conducting perinatal review meetings; (2) contributing to PPIP for more than 10 years; (3) being outside of Cape Town Metro, and (4) demonstrating at least 2 of the following characteristics: team drivers, institutional review, feedback, and communication within the system.^23^ Based on these criteria and stakeholder inputs, 2 PPIP regions were selected, Cape Winelands East and the Overberg (Region 1) and Garden Route and Central Karoo (Region 2), with subdistricts identified within each region. Table 1^34^ provides some context about each subdistrict, noting 2 were within Region 1 and 3 were within Region 2 of the Western Cape.

**TABLE 1.** Description of Western Cape Regional Subdistricts Selected for Study on Implementation of the Perinatal Problem Identification Program^a^

	**Subdistrict A**	**Subdistrict B**	**Subdistrict C**	**Subdistrict D**	**Subdistrict E** ^b^
Approximate catchment area population, No.	95,000	37,500	95,000	93,200	14,400
Annual births,^c^ No.	1,741	506	1,360	1,751	89
Perinatal mortality rate^c^^,d^	11.6	6.0	14.8	17.0	0.0
Facilities in subdistrict^c^	District hospital 5 PHC facilities	District hospital 5 PHC facilities	District hospital 3 PHC facilities	District hospital 5 PHC facilities	District hospital 2 PHC facilities
PPIP region	Region 1	Region 1	Region 2	Region 2	Region 2

Abbreviations: PHC, primary health care; PPIP, Perinatal Problem Identification Program.

^a^ Population data from district reports; births and perinatal mortality rate from PPIP database (accessed March 4, 2022); facility data based on fieldwork observations.

^b^ Classified by the Provincial Department of Health as a “district hospital”^34^ but does not provide surgical services and refers deliveries to another district hospital.

^c^ 2019 data.

^d^ Per 1,000 live births.

Data collection included a systematic document review of relevant policies, guidelines, and literature from South Africa relating to perinatal audit; 56 individual interviews with actors engaged in perinatal audit at different levels of the health system (Supplement 2); administration of a standardized, semistructured questionnaire; review of subdistrict-level PPIP and other relevant documents; and 10 nonparticipant observations of meetings related to the perinatal audit process. National, provincial, district, and subdistrict key informants were purposefully sampled based on their involvement with perinatal audits and included participants with different roles in PPIP and the review meetings (Table 2). Interviews were conducted with at least 10 staff per subdistrict or until saturation had been reached, with the exception of Subdistrict D where only 5 staff were available. Fieldwork and data collection in the subdistricts took place from September 2019 to March 2020, and time spent in each subdistrict varied from 1 half-day to 5 days. Data analysis included thematic content analysis of transcripts and content analysis of relevant documents identified through the desk review. The health policy analysis triangle framework was used to structure the historical mapping and lessons learned.^35^^,^^36^ A tool to measure implementation at the subdistrict and facility levels was also applied (Box 1).^25^^,^^37^^–^^45^

**TABLE 2.** Summary of Key Informants and Meeting Observations in Western Cape, South Africa

**Key Informants** ^a^	**No. (N=56)**
By health system level	
National/provincial	3
Provincial	3
Regional/district	6
Subdistrict	19
Facility	20
Primary health care	5
By subdistrict	
Subdistrict A	10
Subdistrict B	11
Subdistrict C	10
Subdistrict D	5
Subdistrict E	11
Other	9
**Meetings observed (as nonparticipant)**	**No. (N=10)**
Type of meetings	
PPIP provincial meeting	2
M&M meetings	5
M&E meetings	2
Other meetings	1

Abbreviations: M&E, monitoring and evaluation; M&M, mortality and morbidity; PPIP, Perinatal Problem Identification Program.

^a^ A further breakdown of the key informant roles by level is available in Supplement 2.

BOX 1 A Tool to Measure Implementation

A progress-monitoring model and linked tool were developed to assess the implementation of kangaroo mother care^37^^–^^40^ and have been widely used.^41^^,^^42^ The model includes 3 phases: pre-implementation (creating awareness, adopting the concept); implementation (taking ownership, evidence of practice); and institutionalization (evidence of routine integration, evidence of sustainable practice). The linked tool measures tangible inputs for each phase. Belizan et al. theoretically applied the kangaroo mother care implementation model to illustrate themes influencing implementation of perinatal audit in South Africa.^23^

Building from this work, the U.S. Agency for International Development–funded Maternal and Child Survival Program (MCSP) adapted this tool to systematically assess MPDSR implementation across 55 facilities in 4 African countries.^25^^,^^43^ The MCSP tool has been used by others to measure MPDSR implementation in Tanzania and Côte d'Ivoire^44^^,^^45^ and is included in the World Health Organization’s “Maternal and Perinatal Death Surveillance and Response (MPDSR): Materials to Support Implementation.”^7^ For this study, we applied the progress-monitoring model to describe implementation at the national level and used the MCSP tool to measure facility- and subnational-level implementation in the 5 subdistricts.^25^

### Ethical Approval

The ethics approval from the Higher Degrees Committee of the University of the Western Cape was given on November 9, 2018, and approval was received from the Western Cape Provincial Department of Health in July 2019 (NHRD Number: WC_201906_006). Additional information on the methods can be found in Supplement 2.

## RESULTS

Established in the early 1990s in South Africa to capture perinatal mortality data, PPIP identifies modifiable factors to stimulate action as part of a quality-of-care audit cycle.^21^ The program includes 2 primary and complementary components that are implemented at subnational and national levels: (1) the PPIP system and linked tool to help collect and analyze data, and (2) perinatal review meetings (called mortality and morbidity [M&M] meetings). Box 2 provides a brief overview of the history of the South African perinatal audit program.^46^^–^^50^

BOX 2 Brief History of the Perinatal Audit Program in South Africa

South Africa’s history of perinatal audit implementation is broken down according to the 3 phases of the process-model framework: pre-implementation, implementation, and institutionalization.^28^ More details on the history, including a timeline of key milestones, can be found in Supplements 3 and 4.

In Phase 1—pre-implementation (1992–2007)—policy was introduced, ensuring all births and deaths were recorded, including perinatal deaths, and that children had the right to health. Before the perinatal audit program began, different paper-based systems were used by clinicians working in maternity care to identify avoidable factors in perinatal deaths and use data to inform their clinical audit processes. Data from these systems and learning from their application were discussed at the annual Conference on Perinatal Priorities (Priorities Conference), established in 1982, to improve audit systems. One of these paper-based systems was translated into an electronic tool using Microsoft Disk Operating System in 1994 to become the Perinatal Problem Identification Program (PPIP). The tool was refined over the years, using a Windows program in 1999, and lessons and data were shared at the annual Priorities Conferences. The first national meeting on PPIP was held in 2001 to review data from 27 hospitals resulting in the first Saving Babies report.^47^ Thereafter, it grew by word-of-mouth and from people’s interest at the Priorities Conferences, expanding to 244 facilities in 2007.

In Phase 2—implementation (2008–2012)—demonstration of practice, combined with increased political prioritization of neonatal mortality, led to the establishment of a national perinatal review committee in 2008. By 2010, over 80% of all births in the public sector nationally were being entered into the program. By 2012, PPIP expanded from 275 facilities to 588 facilities, with multiple maternal and newborn health programs initiated in response to the findings.^30^ In the “South African Strategic Plan for Maternal, Newborn, Child and Women’s Health and Nutrition 2012–2016,” facilities were encouraged to use PPIP and perinatal reviews became mandatory.^48^

In Phase 3—institutionalization (2012–2019)—the audit program expanded to all facilities with clear instructions on how to implement provided in the “Guidelines for Maternity Care.”^49^ Multiple new programs were implemented in response to the PPIP findings and recommendations, such as Helping Babies Breathe, Management of Sick and Small Newborns, and Essential Steps in Managing Obstetric Emergencies (Supplement Figure S3).^22^^,^^50^ Over 75% of births were recorded through PPIP nationally, indicating widespread use.^29^

The PPIP identifies modifiable factors to stimulate action as part of a quality-of-care audit cycle.

We present the 4 main lessons learned, organized by national level and subnational level from the Western Cape, from initiating, scaling, and sustaining the perinatal audit program in South Africa.

### 1. Integrating the Perinatal Audit Program Into Broader Policy, Guidelines, and Data Systems Embeds the Process Within the Health System

#### National

Before PPIP, South Africa had a national policy for capturing data on all births and deaths (including perinatal deaths) and the right to health for all children.^28^^,^^51^ As PPIP expanded and the review of perinatal deaths became more regular, South Africa incorporated the perinatal audit program into other policy and guidance documents, providing detailed instructions and example tools (e.g., data-capturing forms).^49^^,^^52^ Incorporating guidance in broader national strategies embedded the program into the health system structures. The 2012 policy recognized the value of the PPIP tool for the clinical audit process, which routine data cannot replace.^49^ However, the new 2021 Maternal, Perinatal and Neonatal Health Policy calls for the development of a sustainable surveillance system for maternal, perinatal, and neonatal morbidity and mortality without specific mention of PPIP or linking surveillance to clinical governance structures.^53^ The PPIP data system is not integrated within the routine information system but rather is a complementary, parallel process. The Maternal and Infant Health Care Strategies Research Unit, affiliated with the University of Pretoria, supports the National Department of Health (NDOH) in capturing, analyzing, and summarizing the PPIP data.

#### Western Cape

In the 5 subdistricts assessed, components of the perinatal audit program were embedded and adapted into other guidelines and programs, such as M&M meetings as requirements of the “Ideal Hospital” initiative.^52^ Some participants were aware of NDOH and the Western Cape Department of Health (DOH) guidelines for perinatal audit. Two subdistricts reported receiving standard operating procedures on how to run M&M meetings from district health services. In terms of integrating PPIP into routine data systems, the 2 PPIP regions in the study had 2 different approaches. There was no link between the PPIP data system and the routine information system in region 1; however, in region 2, PPIP data processes were integrated into the system, thus promoting a more sustainable approach.^26^

### 2. Multiple Structures, Along With Continuity of Actors in an Expanding Network, Support Institutionalization

#### National

Multiple national structures support perinatal audit implementation, and these structures coexist, interlink, and rely on each other to function well. The 3 categories of structures include academic, research and training, and governance (Figure), each becoming more formalized over time as the government took over more ownership of the process. First, the Priorities in Perinatal Care Association of South Africa, and their annual conference (Priorities Conference), serves as the academic structure providing a mechanism to share data and learning from perinatal audit as well as provide capacity-building around using and interpreting PPIP and related audit findings.^54^ Participants receive continuous professional development points for attendance and primarily receive funding from their workplace or the provincial DOHs to attend.

National structures within academic, research and training, and governance categories support perinatal audit implementation and coexist, interlink, and rely on each other to function well.

**FIGURE.**
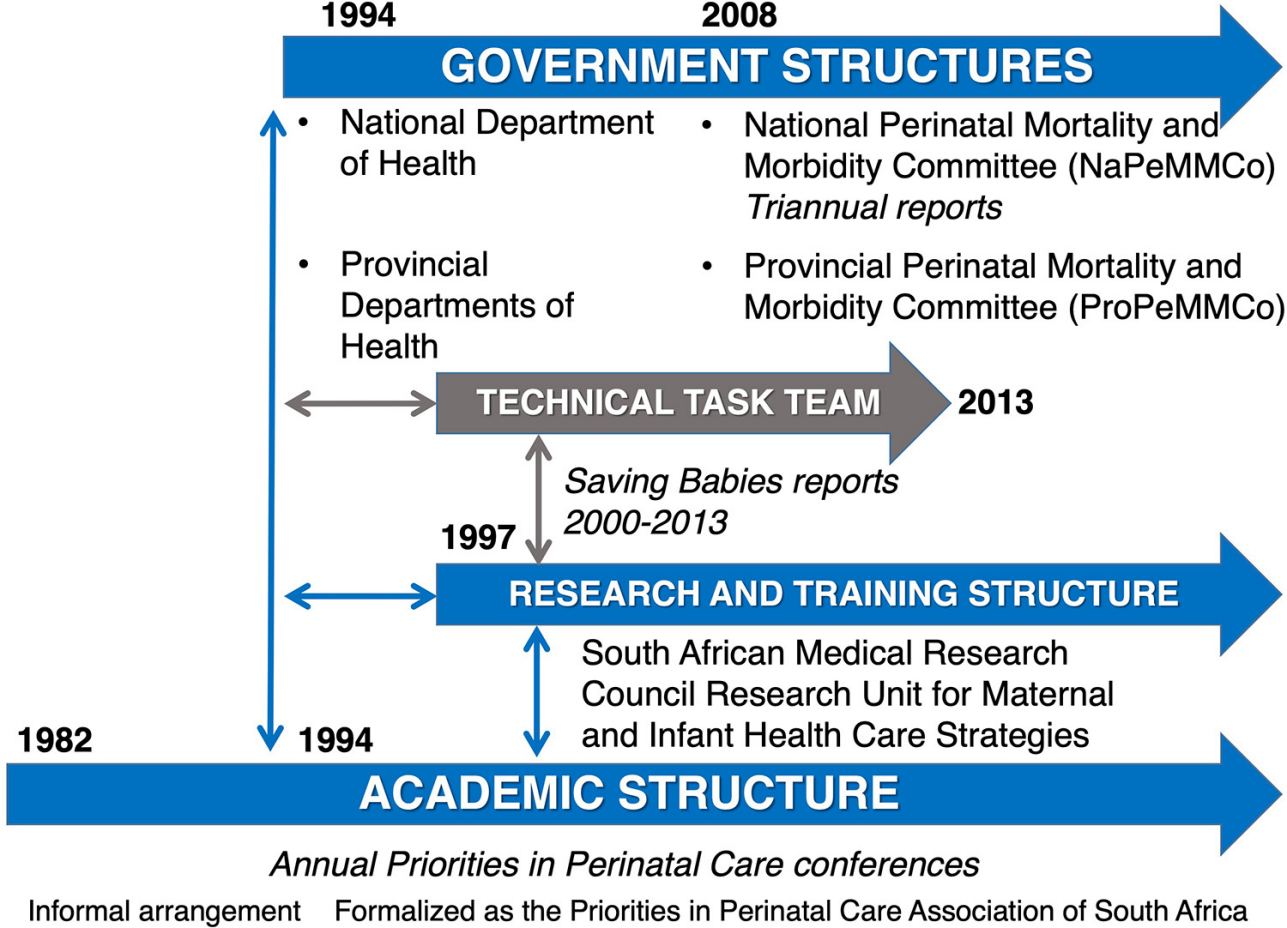
National Structures Supporting Implementation of the Perinatal Audit Program in South Africa

Second, the research and training structure provides continuous oversight and implementation support. The Maternal and Infant Health Care Strategies Research Unit, established in 1997, has overseen the administrative and technical aspects of PPIP. By becoming an official extramural unit of the South African Medical Research Council, the Unit gave NDOH ownership of the process. The Unit evolved over time and continues to support these PPIP-related activities.

Finally, the governance structures include the NDOH and provincial DOHs and their related perinatal review committees. Initially, a Saving Babies Technical Task Team was established to support implementation of PPIP with diverse membership, including NDOH, and funded initially by the South African Medical Research Council/University of Pretoria unit. When the Ministerial National Perinatal Mortality and Morbidity Committee (NaPeMMCo) was established in 2008, it formalized this structure within NDOH. The committee meets on a semiannual basis, reports to the NDOH annually, and produces a publicly available triennial report.^29^^,^^30^^,^^55^^,^^56^ NaPeMMCo recommended each province also have a perinatal review committee, with 1 provincial coordinator for PPIP on the national committee to ensure a coordinated approach of data flow and analysis.^56^ NDOH and the provincial DOHs fund related costs for these meetings (e.g., travel).

These structures are linked by the actors who engage in the processes, with individuals often engaged in more than 1 structure. A continuity of actors in these structures with a core set of PPIP champions at the helm,^28^ as well as the expansion of the network with new actors emerging and taking more leadership roles, promoted institutionalization. Many of the provincial and regional PPIP actors remained in their roles for long periods of time. For example, the provincial and regional PPIP coordinators in the Western Cape interviewed had been in their roles for more than 10 years and had established systems to support and use the perinatal audit program to strengthen clinical practice, allowing predictability in communications, engagement, and expectations from actors at the subdistrict and provincial levels. However, recent transitions of core actors to the perinatal audit program at all levels and within NDOH, either through new posts or retirement, have raised concerns about the sustainability and quality of the program.

*I don’t think anybody will ever be him. Or be able to replace him. [Others] they’re not the same. They just fill in the forms and don’t really teach us. With Dr. X, you are always learning.* —Family physician

A continuity of actors in these structures with a core set of PPIP champions at the helm promoted institutionalization.

The range of actors has been multidisciplinary. The Priorities Conference particularly emphasizes and encourages the involvement of a range of health professionals, including health workers, managers, academics from different disciplines, national and provincial DOH staff, and implementing partners. The inclusive nature of this structure was especially important at the start, given the political and historical context in the country.

*It was the only meeting in South Africa where there was no hierarchy - midwives, nurses, doctors, MOs [medical officers], consultants - they were all at 1 level. And that in the early [19]90s was a major thing. Because remember it was white - and they were the doctors - and the blacks were the nurses.* —Subnational stakeholder

#### Western Cape

Bergh et al. unpacked the complex web of structures supporting implementation of perinatal audit at the subnational level, notably provincial, regional, and subdistrict levels (Supplement 5).^27^ These structures are essential for communication and flow of data and information between and within levels,^22^ and similar structures exist in the Western Cape. The Western Cape Provincial Perinatal Mortality and Morbidity Committee meets semiannually, during which regional PPIP coordinators present the data from their subdistricts to inform provincial health system planning. The provincial PPIP coordinators are responsible for compiling the data and recommendations for the province and sending the results to the national level. Each subdistrict has regular perinatal review meetings, whereby clinical staff, management, and other actors working in maternity care meet to review and discuss perinatal deaths. The involvement of both managers and clinical specialists (family physician, obstetrician, and/or pediatrician) and other health workers enable accurate analysis of the cases and identification of related and practical recommendations. Within each subdistrict, an informal team of actors implements perinatal audit with different people leading different parts of the process (Supplement 6). These informal teams demonstrate shared commitment among actors and the importance of multidisciplinary engagement.

*I think this is a program that you need to drive with a team of different departments. If you have a doctor, you have the operational manager of maternity, we have a sister maybe, we have a clerk.* —Information officer

The PPIP system (i.e., forms, software, and reporting system) is used to identify, report, and analyze deaths to inform the quality-of-care audit process. The flow of information starts with capturing data in the registers and in a designated data-capturing form; the data is then entered into the PPIP electronic system and sent to the regional PPIP coordinator, who then sends the data to the provincial PPIP coordinator as well as presents the data at provincial perinatal review meetings.^22^ In some subdistricts, PPIP data are analyzed and presented at the monthly subdistrict and quarterly district monitoring and evaluation meetings, as well as the M&M meetings. Feedback loops are in place to share recommendations and actions with different teams and levels through existing meetings, communication channels, and other clinical governance structures.

*Say we need education … or equipment… selective things will go through to management meeting and we'll discuss it there and from there on it will be our responsibility.* —Clinical manager

### 3. Intentional and Continuous Demonstration of Impact, as well as Local Adaptation, Are Essential for Buy-In and Ownership to Sustain Practice

#### National

Intentional efforts were made at the start with PPIP to demonstrate impact, engage a diversity of stakeholders, and embed the process within NDOH in an effort to get buy-in. The first Saving Babies report (2000) was a product of a workshop held to collate data, identify areas of concern, and collectively make recommendations.^47^ The first workshop was multidisciplinary and inclusive.

*The delegates came from throughout South Africa and for once the meeting was not dominated by academics or administrators, but by the health workers from the coalface.* —Saving Babies Report 2000^47^

This model of inclusive engagement to develop the Saving Babies reports continued as the number of participants and facilities presenting data from PPIP expanded (Box 2).^57^^–^^64^ The annual engagement through the Priorities Conferences and other PPIP-related workshops ensured buy-in after the initial phase and enabled continued sharing of local adaptation and experiences during the implementation and institutionalization phases. However, there has not been a specific workshop since 2009. The Saving Babies reports continued until 2016,^30^ and no report has been published officially since, though NaPeMMCo has continued to submit a triennial report to the NDOH.^29^

Rhoda et al. map the new initiatives that have been rolled out nationally from 2008 in response to the perinatal audit program (Box 2, Supplement Figure S3).^50^ Over the years, these new and existing programs were promoted in the NaPeMMCo reports, discussed and presented at the Priorities Conference, and taught as part of training for quality improvement, furthering dissemination and buy-in. Clear communication on data and actions has been an important contribution, influencing the scale-up and sustainability of PPIP.

Clear communication on data and actions has been an important contribution influencing the scale-up and sustainability of PPIP.

*I think there’s been a lot that has come out of just the simple clear messages from PIPP through the Saving Babies [reports]… being implemented or at least being taught at academic level and training level.* —National stakeholder

In terms of direct impact on mortality, the evidence is inconclusive.^65^^–^^67^ The largest study from South Africa investigated perinatal mortality across 163 facilities using the perinatal audit program over 5 years and found wide variation in mortality changes.^66^ Poor quality of implementation of the program (e.g., not identifying appropriate modifiable factors) may have resulted in the increased mortality rates in some settings, but further research is needed to compare the quality of practice linked to outcomes between facilities in South Africa.

#### Western Cape

Swartz et al. speak to the value proposition of PPIP as a tool to help present data and motivate for change based on the data resulting in high levels of buy-in from health workers and managers.^28^ The Western Cape research confirmed that buy-in increases when people see results from the perinatal audit program. At the subdistrict level, most participants could give examples of how change occurred due to perinatal audit and believed it helped to improve service delivery.

*If there’s equipment situation, they want to identify it through the PPIP. Or we need more staff. Then they got extra 2 sisters for labor ward and we got like more CTG machines.* —Operational manager of maternity ward

*I think it does have an impact… there’s been a few M&M’s where I’ve actually written SOPs to change practice and we’ve implemented it.* —Medical officer

At the same time, some participants felt that they were not informed about how the PPIP data was used and what impact the process had. Subdistrict managers did not routinely report back actions at perinatal review meetings, and some meeting minutes did not include actions (Supplement 6). Because some actors would only input on the PPIP form or report the data and not hear about the actions taken forward at the subdistrict level, participants from across levels (primary to referral) questioned the purpose of the process.

*We do all of this work [data collection, analysis, reporting] for nothing; nothing changes.* —Outreach specialist

*The stuff that they talk about [at M&M meetings] I don’t actually know if they implement it because it’s more medical related. But from an administrative point of view it hasn’t improved.* —Subdistrict information officer

*If it was more emphasized on why we are doing it - to see results, to see where we're lacking - I think then people might have more of an input in it.* —Primary health care nurse

Another aspect related to buy-in is local adaptation, which has been well documented by studies in South Africa.^22^^,^^23^ In the Western Cape, the subdistricts had similar yet adapted processes. Table 3 describes the process in each subdistrict according to the MPDSR cycle,^7^^,^^11^ with factors supporting sustained practice as well as distinctions between sites. Common inputs included PPIP focal points, standard reporting forms, and regular review meetings, but there are variations in who did what, which forms were used, and how often meetings took place (Supplement 6). The subdistricts adapt the perinatal audit process to their contexts, actors, and structures for it to work. At the same time, the consistency of the higher-level structures, such as the provincial-level meetings or national requirements, held subdistricts accountable to practice perinatal audit.

**TABLE 3.** Descriptive Factors Enabling Sustained Practice of the Perinatal Problem Identification Program in Western Cape, South Africa

**Dimension/Question**	**Main Finding**	**Common Factors Across Sites**	**Distinctions Between Subdistricts**
Identification and reporting: How do people identify and report deaths?	People identify and report deaths on regular bases because they have a standard reporting system and PPIP regional focal points monitor data inputs.	Standard reporting forms available. Standard reporting software/mechanism available (PPIP software). Follow-up by PPIP coordinators at provincial and regional levels to ensure data is collected and submitted.	Different reporting forms used after a death. Information officer responsible for PPIP data collection, capturing, and reporting working with doctors and operational manager (C, D, E). PPIP data collection, capturing, and reporting rely on clinicians, the nursing manager, and operational managers (A, B).
Reviewing deaths: How do people review deaths?	Review meetings take place regularly as part of national requirements with multidisciplinary engagement, although the meeting process varied between cases.	Facilities are required to do a minimum number of review meetings each year (10), and perinatal-focused meetings are counted towards this requirement. Multidisciplinary engagement. Outreach specialist (obstetrician or pediatrician) attends review meeting.	Meetings are scheduled monthly (A, B). Ad hoc review meetings (C). Multiple meetings related to review process (D). Facilitation by hospital staff (C, D, E). Outreach specialist facilitates meeting (A, B). Outreach specialist attends and contributes during the meeting (C, D).
Analysis and recommendations: How do people analyze data and make recommendations after perinatal death?	Data analysis and use for decision-making varied between cases but all data is used for planning at provincial level.	Involvement of managers and clinical specialists (obstetricians, pediatricians, and/or family physicians) in review meeting to analyze cases and identify relevant and feasible recommendations. Regional PPIP focal person conducts analysis of data for the region and makes recommendations to provincial level during biannual meeting.	Data analysis by information officer (C, D, E). PPIP data used at M&E subdistrict meeting to make recommendations (C). PPIP data used at the quarterly M&E district meeting for health system planning (C, D, E).
Response and actions: How do people respond to the recommendations and take actions forward after perinatal death?	Subdistrict management teams oversee response and actions. Feedback loops for sharing information are in place.	Management oversees implementation of actions. Feedback loop in place to share with different teams and levels.	No formal follow-up (A, B). Formal follow-up by QA manager (C, D). PPIP data used at the M&E district meeting for health system planning (C, D, E) and M&E subdistrict meeting (C).

Abbreviations: M&E, monitoring and evaluation; PPIP, Perinatal Problem Identification Program; QA, quality assurance.

### 4. Institutionalization Is a Continuous Process, Not a Destination

#### National

The perinatal audit program started nearly 3 decades ago and has continuously evolved and expanded (Box 2). The initial expansion of PPIP from 27 to 244 facilities took 15 years because the program was entirely voluntary. Once the program became more official with the establishment of the national committee, the number of facilities reporting more than doubled to 588 in 4 years. After PPIP became formally embedded in national policy and guidelines, 75.8% of deliveries recorded through the routine health information system were also reported to PPIP in 2012–2013.^57^ There was an increase to 83.9% of deliveries reported in 2014–2016^30^ but a decline to 75.8% of deliveries in 2017–2019,^29^ demonstrating widespread practice but not yet complete coverage of the perinatal audit program. The chronology of this national program (Supplement 4) demonstrates how long it can take for the introduction and scale-up of MPDSR. The declining coverage of deliveries reported to PPIP in recent years also signals that backsliding is possible without continuous efforts.

The recent declining coverage of deliveries reported to PPIP signals that backsliding is possible without continuous efforts.

#### Western Cape

To move beyond coverage as measured by number of deliveries reported to PPIP, we used a tool adapted by the U.S. Agency for International Development–funded Maternal and Child Survival Program (MCSP) to understand coverage of practice using tracer indicators (Box 1; Supplement 6).^25^ All 5 subdistricts achieved the status of institutionalization, with a median score of 24.21 out of 30 (Table 4; Supplement 6). Progress markers within the pre-implementation phase were achieved across the subdistricts, but there was some variation between subdistricts on achieving progress markers in the implementation phase. Nonetheless, most progress markers were achieved in the implementation and institutionalization phases across the subdistricts, such as use of tools, meeting minutes, regular meetings, multidisciplinary engagement, and evidence of change from the review process. Missing elements that held back a complete score across all cases included budget allocation, reporting findings and progress to community, and related training in the past year. Additional progress markers not fulfilled in 2 or more cases included a code of conduct, follow-up actions recorded in minutes, orientation, data trends displayed, and plans for related training (Table 4). The high scores achieved were to be expected given these subdistricts were selected because they had a sustained practice of perinatal audit.

**TABLE 4.** Meeting the Progress Markers for Each Stage of Implementation of the Perinatal Problem Identification Program in Western Cape, South Africa

**PhaseImplementation Stage**	**Progress Marker**	**Score for Progress Marker**				
		**Subdistrict**				
		**A**	**B**	**C**	**D**	**E**
Pre-implementation						
1. Creating awareness (2 points)	Awareness by management (1 point)	1	1	1	1	1
	Committed leader(s) (1 point)	1	1	1	1	1
2. Adopting the concept (2 points)	Conscious decision to implement (1 point)	1	1	1	1	1
	Committee formed (1 point)	1	1	1	1	1
Implementation						
3. Taking ownership (6 points)	Tools available (1 point)	1	1	1	1	1
	Tools include cause of death (1 point)	1	1	1	1	1
	Tools include modifiable factors (1 point)	1	1	1	1	1
	Tools include place to follow up on actions taken (1 point)	1	1	1	1	1
	Understanding of process for conducting meetings (0.5 point)	0.5	0.5	0.5	0.5	0.5
	Staff meeting conduct agreement available (0.5 point)	0	0	0.5	0.5	0.5
	Budget or support to conduct death reviews (1 point)	0	0	0	0	0
4. Evidence of practice (7 points)	Meeting minutes available (1 point)	1	1	1	1	1
	Meeting minutes include action items (1 point)	1	1	1	1	1
	Meeting minutes include follow-up from previous meetings (1 point)	0	1	1	0	0
	Meeting notes respect confidentiality of staff and patients (1 point)	1	1	1	1	1
	Face-to-face or written orientation to death reviews (1 point)	1	0	0.5	0.5	0
	Data trends displayed or shared (2 points)	2	1	2	2	0
Institutionalization						
5. Evidence of routine integration (7 points)	Evidence of change based on recommendation (3 points)	3	3	3	3	2
	Death review meetings are held at stated interval (e.g., weekly, monthly) (1 point)	1	1	1	1	1
	Multidisciplinary engagement (2 points)	2	2	2	2	2
	Evidence of reporting findings and progress to community (1 point)	0	0	0	0	0
6. Evidence of sustainable practice (6 points)	Over 1–2 years of ongoing practice (2 points)	2	2	2	2	2
	Plan in place to ensure all staff receive MPDSR training (1 point)	1	0	0	0	0
	Evidence that staff have received MPDSR training in the past year (1 point)	0	0	0	0	0
	Score on the first 5 constructs (divided by 12)	1.71	1.63	1.79	1.71	1.42
	Total (30 points)	25.21	23.13	25.29	24.21	20.42

Abbreviation: MPDSR, maternal and perinatal death surveillance and response.

The failure to achieve some progress markers may indicate issues with the predetermined factors in the MCSP tool itself rather than lack of institutionalization. This may suggest that some components are either not essential for sustained practice or that the framing of these components needs reconsideration (Supplement 6). For example, subdistricts did not have direct budget allocation to support the perinatal audit program; however, the staff time to participate in the related activities was recognized as part of their work and existing salary.^26^ Feedback to the community did not seem necessary for continuous practice. In fact, participants felt the M&M meetings should be for clinical staff only and that direct involvement of the affected family in the perinatal audit was highly sensitive. The lack of continuous tracking of staff, however, may be an issue given the gaps in the quality of practice, such as lack of actions in meeting minutes (Supplement 6).^26^

## DISCUSSION

Lessons from South Africa’s experience of implementing a perinatal audit program for nearly 3 decades reveal enabling factors, such as core structures, as well as vulnerabilities to sustainability. The results show that multiple and evolving national and subnational structures benefited from ongoing intentional efforts to establish and nurture a network of diverse actors. Local adaptation and demonstration of impact helped to ensure buy-in and ownership initially, although feedback has not been continuous at all levels of the system. The integration of the perinatal audit program activities in national policy and guidelines embedded it within the health system until recently. Finally, examining implementation in settings with sustained practice through multiple approaches revealed shortcomings in both practice and in the tools developed to measure sustained practice.

Local adaptation and demonstration of impact helped to ensure buy-in and ownership initially although feedback has not been continuous at all levels of the system.

Perinatal audit is being expanded in LMICs as part of MPDSR and promoted by the World Health Organization and partners as a combined approach.^7^ The PPIP tool has also been adapted and used in other settings outside of South Africa.^68^^–^^70^ Box 3 provides 10 lessons from this research, which may help South Africa and other countries in their efforts to introduce, expand, and sustain perinatal audit. These lessons relate to findings from other studies examining the history of implementation of MPDSR processes.^3^^,^^71^^–^^73^ More operational research and in-depth studies, especially from LMICs, will be needed to better understand implementation history and practice in different contexts.^71^^,^^74^

BOX 3 Ten Lessons From Sustaining a Perinatal Audit Program in South Africa

1. **Ensure ownership by national and subnational governance structures by integrating perinatal audit into broader health policies, guidelines, and routine information systems.** For scale-up and sustainability in South Africa, it helped to include perinatal death audits and the Perinatal Problem Identification Program (PPIP) in national plans and related initiatives to explain value add (e.g., extra data elements not captured in the routine health information system to measure quality of care), support implementation, and track coverage. At the subnational level, including PPIP in annual district health plans and reporting embeds the process into budgets and monitoring and evaluation for accountability.
2. **Set up formal and functional structures at all levels of the health system (facility, subnational, and national) that oversee and coordinate implementation, including structures that support governance, research, and training.** For South Africa, informal structures formalized over time with continuous engagement from the National Department of Health to ensure buy-in and ownership.
3. **Engage relevant stakeholders at all levels of the health system in the collection, analysis, and reporting of data and recommendations/response and include these activities as part of their job descriptions.** In South Africa, the multidisciplinary engagement was intentional from the start and occurred at multiple levels of the system (i.e., facility perinatal review meetings as well as at subnational/national level for data analysis and development of national reports). In addition, integrating activities related to the perinatal audit process as part of job descriptions and performance monitoring supports sustainability.^26^ The role of the community in facility-based perinatal audit requires contextual consideration and more research.^13^
4. **Enable and encourage local adaptation of perinatal audit processes across the steps of the audit cycle (e.g., who does what, when meetings occur, and how information is shared) to support accountability, sustainability, and ownership.** While the core elements of data capture and reporting and death review meetings took place, the implementation processes varied across the 5 subdistricts, signaling that there is no “one-size-fits-all” model. Common implementation processes were observed between the subdistricts within the 2 PPIP regions reflecting the leadership of the regional PPIP coordinators. Provincial and regional oversight of senior health professionals, who were officially mandated to improve care at the local level, drove accountability at the local level.
5. **Share implementation experiences and learn from each other.** South Africans engaged with PPIP have had opportunities at different levels of the health system to meet on a regular basis to share implementation experiences (e.g., lessons, challenges, innovative solutions, and capacity development). At the national level, the annual Perinatal Priorities Conference has provided a platform for users to engage regularly. The perinatal review meetings provide this platform at a subdistrict and provincial level.
6. **Demonstrate impact of perinatal audit at all levels of the health system.** Nationally, regular demonstration of practice through the Saving Babies reports and workshops encouraged others to engage and buy into the process. Clear communication around the importance and value of the perinatal audit program has also helped. Subnationally, evidence of change due to the perinatal audit process encouraged participation.^26^ Reporting back actions or “the response” during the next perinatal review meeting can help garner buy-in by sharing the benefits of the perinatal audit program.
7. **Recognize that scale-up and institutionalization take time and plan accordingly.** The World Health Organization guidelines on maternal death surveillance and response provide helpful guidance on how to start small and expand over time.^11^ The perinatal audit program in South Africa started nearly 3 decades ago, and intentional efforts and investments were made to embed the process within the health system.
8. **Continuously advocate for implementation to ensure perinatal audit remains in policies, programs, and practice.** The expansion of the perinatal audit program in the 2000s demonstrates what can be achieved with intentional efforts by champions.^28^ Yet, the recent decline in coverage of PPIP usage and exclusion of PPIP from the new maternal and newborn health policy signals the fragility of such programs and the need for continuous efforts to sustain them.
9. **Monitor coverage and implementation practice at all levels of the system.** South Africa has done well to ensure regular reporting of PPIP data in the national triennial reports, including coverage of deliveries reported through PPIP. The Maternal and Child Survival Program tool is 1 mechanism to monitor practice at the facility level. The tool enables users to conduct a brief assessment of coverage but potentially misses some elements that may be relevant across contexts (e.g., organizational culture) and other elements that may require more nuanced contextualized understanding (e.g., community engagement).
10. **Conduct more research on impact and quality of perinatal audit.** The studies from South Africa assessing impact on mortality and implementation factors reveal inconclusive and often inconsistent findings.^21^^–^^23^^,^^27^^,^^28^^,^^75^ Maternal and perinatal death surveillance and response, including perinatal audit, is a complex social process involving many steps and people, engagement at multiple levels, and linkages to other clinical governance and quality improvement activities.^13^ More research across diverse epistemologies and at different levels will be needed for better understanding of implementation across different settings.

Factors influencing implementation of MPDSR in LMICs have been identified and examined in the literature,^13^ including for South Africa.^23^^,^^28^^,^^75^ This article adds to that body of literature showing that many factors influencing institutionalization may not be easily quantified or measured (i.e., networks, team dynamics, and individual motivation).^13^^,^^23^^,^^26^^,^^27^ Institutionalization of the South African perinatal audit program has been supported by mainstreaming it into national policies and guidelines, demonstration of practice, local adaptation, and continuity of actors. Academic and technical structures, linked to NDOH, as well as synergies with other quality improvement and clinical governance structures, also supported practice.^76^ Subnationally, this article highlights that different actors took on various roles and tailored the process to their context, reflecting the importance of local adaptation, a well-known core element of sustainability.^77^^–^^80^ Currently, global and regional surveys monitoring MPDSR do not capture all the structures that may be needed to institutionalize practice, such as academic and technical structures, and do not include other factors, such as local adaptation.^81^

For South Africa, the future of this perinatal audit program cannot be assumed to be indefinitely sustainable or perfect in its current form despite its legacy and widespread coverage. The gaps in the new policy of linking MPDSR to the audit cycles and clinical governance activities^53^ and the failure to publish the recent Saving Babies report^29^ raise concerns about the government’s understanding, buy-in, and ownership of the perinatal audit program.

Additionally, the scale of the perinatal audit program has dipped in coverage, and there are observed quality gaps even amongst facilities with long histories of practice.^26^^,^^75^ The stagnant perinatal mortality rates combined with reduced PPIP coverage are worrisome, especially in the absence of an alternative tool that can help clinicians and managers assess their maternal and perinatal health outcomes. Audit and feedback aim to improve professional practice through identifying local problems and solutions,^82^ and currently, PPIP is the only tool nationally available to help clinicians and managers collect and analyze their maternal and perinatal health data for audit. Reasons for the policy and implementation gaps will require further investigation.

The uncertainty of impact of the current perinatal audit program on mortality reduction in South Africa^21^ does not necessarily indicate failure of the program, especially when global systematic reviews on the impact of MPDSR rest on too few studies.^73^^,^^74^^,^^83^ Quality of practice or “functional PPIP” is necessary for health system improvements.^22^ A complementary article of this research presents evidence that there are other benefits to the perinatal audit program beyond mortality outcomes, such as skills development, individual and collective motivation, and improved teamwork and dynamics.^26^ Users and policymakers need to consider and measure the impact of the whole process rather than only 1 component of the complex MPDSR process. Consideration of integrating PPIP within the routine health system should include lessons from this research and will require additional research to assess potential in other contexts.^26^ Tracking other forms of outputs, such as documenting success stories and feedback and demonstrating data use for decision-making, may enable managers and policymakers to see the value-add of MPDSR beyond mortality outcomes.^23^^,^^26^^,^^75^ As with any quality improvement intervention, continuous activities and linked improvements are needed to sustain and strengthen practice.^66^^,^^74^^,^^83^^,^^84^

Users and policymakers need to consider and measure the impact of the entire MPDSR process, rather than only 1 component of it.

Use of the MCSP progress-monitoring tool alongside other study methods showed gaps in the implementation process as well as gaps in the tool itself. Some of the progress markers not achieved in this study, such as budget to support the reviews, community engagement, and training, were also not achieved in other African settings,^25^ suggesting the need to reconsider the relevance of these progress markers. Application of the MCSP tool may help researchers and program managers evaluate if MPDSR activities are taking place (coverage),^23^ but for the most part, it is not able to measure how well it is practiced (quality). For example, committees can be in place, but if perinatal review meetings are not well facilitated, it can lead to a blame culture, which can derail the implementation process.^85^ Strategies to implement a positive implementation culture have been identified, and more research on the quality of practice is needed.^85^ Just as the original tool designed for kangaroo mother care has evolved,^37^^,^^38^^,^^86^ the MCSP tool may require adaptation to better measure implementation coverage and quality of practice.

Understanding sustainability requires qualitative research of the national and subnational structures, their history of origin, ownership, and relationships among actors within and between these structures.^23^^,^^27^^,^^28^^,^^75^ To further advance implementation at all levels, we will need more nuanced health policy and systems investigations about what drives and motivates those who are initiating and overseeing implementation and how to create a culture of adaptive learning through MPDSR that supports trust, communication, and collaboration over time.^87^

### Limitations

This study focused on understanding implementation at the national level and subnational levels. The 1 province and 5 subdistricts were selected based on criteria that enabled contexts where we could describe and examine sustained implementation of perinatal audit. The case study research can only speak to the 5 subdistricts and may not be generalizable across South Africa or even the Western Cape. It should also be noted that the Western Cape has a unique health system history and approach in South Africa with demonstrated strong leadership, innovative and functional management processes, and multisectoral engagement.^88^^–^^90^ Broader health system enhancements may have influenced the implementation of the perinatal audit program in these subdistricts.

This study collected information on perinatal audit, which is a sensitive topic given the nature of exploring adverse incidents by reporting data on deaths as well as reviewing the situation surrounding the death. Through individual interviews, this study included perspectives from a wide range of stakeholders; however, not all stakeholders were included due to lack of availability or data saturation. Participants may have reserved their true opinions about the process or experience and may have changed their behavior during the observed review meetings. Data collection stopped at the end of March 2020 due to the COVID-19 pandemic and related restrictions. This, unfortunately, prevented further data collection, including observation of additional meetings and timely validation meetings with the subdistricts.

To ensure rigor and trustworthiness, triangulation of the different data sources was used to verify and validate information, including field notes, observations, and follow-up interviews with specific people. There was possible interpretive bias of the doctoral candidate (MK) and other authors given their involvement in the development and adaptation of the progress-monitoring tool and involvement in the perinatal audit program. Efforts were undertaken to minimize bias, such as the use of a semistructured interview guide, a standardized tool, thematic content analysis applying an analysis coding framework, and validation with multiple stakeholders and sources.

## CONCLUSION

The institutionalization of the perinatal audit program in South Africa provides some rich lessons that may be helpful to stakeholders in the country and in other countries that seek to expand and strengthen MPDSR. Key factors supporting sustained practice include national and subnational structures that evolve and enable routine flow of information to all levels of the health system and continuously provide formal touch points among actors to share learning and information about practice. Enabling local adaptation of the intervention process at subnational levels whilst also having clear national policies and guidelines in place for reporting and tracking progress promotes sustainability, but this requires continuous efforts to keep the program in policy. The national policy and implementation gaps related to PPIP in South Africa signal the need for continuous efforts to sustain the program and improve the quality of practice. To monitor the uptake and sustainability of these programs, we need to go beyond tracking measurable or tangible inputs necessary for implementation to include research approaches that allow us to explore the importance of contextual, local adaptation, and underlying issues that support sustainability in more intangible but critical ways.

## Supplementary Material

GHSP-D-22-00213-supplements.pdf

## Notes

**Peer Reviewed**

**First published online:** March 3, 2023.
