# Supplementary material for: From Pre-Implementation to Institutionalization: Lessons From Sustaining a Perinatal Audit Program in South Africa
Source: Glob Health Sci Pract. 2023 Apr 28;11(2):e2200213. doi: 10.9745/GHSP-D-22-00213 (PMC10141437; doi:10.9745/GHSP-D-22-00213)
Supplement: GHSP-D-22-00213-supplements.pdf [file GHSP-D-22-00213-supplements.pdf]

Supplement to: Kinney MV, George AS, Rhoda N, Pattinson RC, Bergh AM. From pre-implementation to institutionalization: lessons from sustaining a perinatal audit program in South Africa. *Glob Health Sci Pract*. 2023;11(1):e2200213. https://doi.org/10.9745/GHSP-D-22-00213

Supplement Contents

Supplement Figure S1. PPIP regions in the Western Cape, South Africa 1

Supplement 2. Methods additional information..3

Supplement 3. Desk review additional information 7

Supplement 4. Policy analysis 14

Supplement 5. Provincial and subnational structures 21

Supplement 6. Case study research additional information..22

SUPPLEMENT 1: OVERVIEW OF PERINATAL PROBLEM IDENTIFICATION PROGRAM (PPIP) STRUCTURE IN THE WESTERN CAPE

The Western Cape Province, South Africa is divided into 1 metropolitan municipality (City of Cape Town Metropolitan Municipality) and 5 district municipalities (West Coast, Central Karoo, Garden Route, Overberg, and Cape Winelands), which are further subdivided into 24 local municipalities. Specific to PPIP, there are also 5 regions (Cape Metro East, Cape Metro West, West Coast and Cape Winelands West, Cape Winelands East and Overberg, Garden Route and Central Karoo). The PPIP regions do not align with the district or sub-districts municipal borders but rather link to the Regional Referral Hospitals. Each Regional Referral Hospital designates a regional PPIP coordinator, who is often a specialist (e.g. obstetrician or pediatrician), to oversee implementation of perinatal audit in the sub-districts of which they oversee as an outreach specialist. Figure S1 shows a map of the Province with the PPIP regions overlaid with the districts and sub-districts.

Supplement to: Kinney MV, George AS, Rhoda N, Pattinson RC, Bergh AM. From pre-implementation to institutionalization: lessons from sustaining a perinatal audit program in South Africa. *Glob Health Sci Pract*. 2023;11(1):e2200213. https://doi.org/10.9745/GHSP-D-22-00213

FIGURE S1. MAP OF PPIP REGIONS IN THE WESTERN CAPE, SOUTH AFRICA, OVERLAID WITH DISTRICT AND SUB-DISTRICT LEVEL MAP OF WESTERN CAPE PROVINCE, SOUTH AFRICA

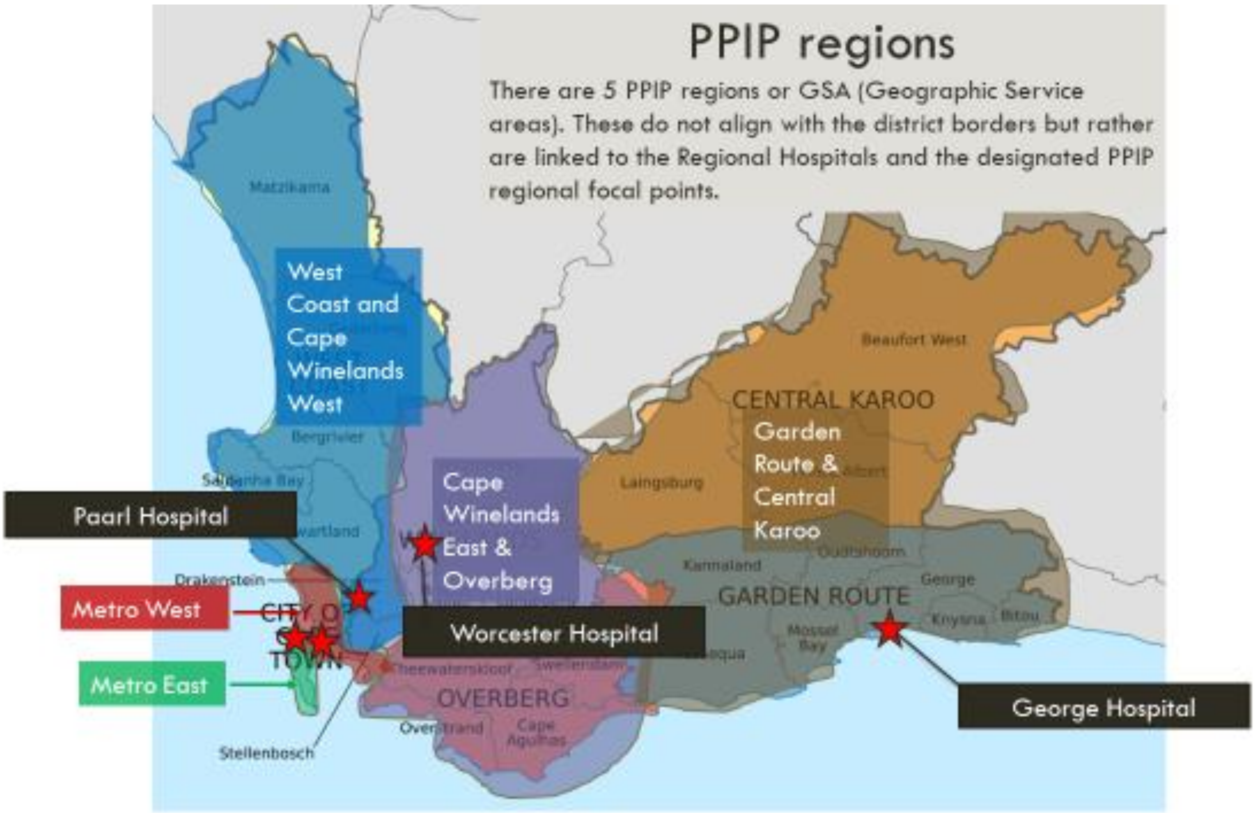

★ Red star signifies Regional Referral Hospital

Source: Mlotshwa M, Smit S, Williams S, Reddy C, Medina-Marino A. Evaluating the electronic tuberculosis register surveillance system in Eden District, Western Cape, South Africa, 2015. Glob Health Action. 2017;10(1):1360560. doi: 10.1080/16549716.2017.1360560.

Supplement to: Kinney MV, George AS, Rhoda N, Pattinson RC, Bergh AM. From pre-implementation to institutionalization: lessons from sustaining a perinatal audit program in South Africa. *Glob Health Sci Pract*. 2023;11(1):e2200213. https://doi.org/10.9745/GHSP-D-22-00213

SUPPLEMENT 2. METHODS ADDITIONAL INFORMATION

Desk review

To understand the context and history of implementation, MK conducted a desk review of related national guidelines, conference proceedings and literature on implementation of the perinatal death audit program in South Africa. A content analysis was undertaken applying a health policy analysis triangle framework.[1,2] For content, national policies, guidelines and reports were mapped chronologically and reviewed and information on perinatal audit extracted. For actors, the names of authors on the perinatal audit related reports were extracted and mapped by year, province and role. For process, activities related to the response of perinatal audit were extracted from the perinatal audit related reports and Conference for Perinatal Priorities proceedings and mapped chronologically. For context, data on the number of facilities implementing PPIP was extracted from the perinatal audit related reports and mapped chronologically. MK conducted all extraction and mapping and verified data with national stakeholders.

Case study research of sustained implementation in the Western Cape

A multiple case study design was applied to understand the "how" or "why" of sustained implementation.[3] We used a multiple holistic design whereby the sub-district was considered as a unitary whole allowing for comparison across settings to gain insights on factors influencing sustained implementation of perinatal audit.

We also set out to systematically assess implementation of the perinatal audit program in five sub-districts in the Western Cape, South Africa, applying a standardised scoring methodology. Qualitative and quantitative data collection methods were employed, including observations (e.g., onsite review of facility documents) and semi-structured key informant interviews with subnational and facility managers and staff.

Sampling

The PPIP reporting structure in the Western Cape comprises five PPIP regions, which are aligned to the regional hospitals with a designated regional PPIP coordinator who oversees implementation. The district level-one hospitals manage all of the deliveries in a sub-district, unless referral is required. Antenatal and postnatal care services take place at the primary health care (PHC) level. Perinatal audit considers the full continuum of care and engages both hospital and PHC staff; therefore, each case is defined as a "sub-district" with the district hospital as the host of the process. Criteria for sub-district selection included: 1) currently conducting perinatal review meetings; 2) contributing to PPIP for over 10 years; 3) a district hospital outside of Cape Town Metro, which has a unique system;[4] and 4) demonstrating at least two characteristics from a previous study on perinatal audit in South Africa: team drivers, institutional review, feedback and communication within the system.[5] Based on these criteria and stakeholder inputs, two PPIP regions were selected, Cape Winelands East and the Overberg (Region 1) and Garden Route and Central Karoo (Region 2) and then two sub-districts identified within each: case A and B in Region 1; and case C, D and E in Region 2.

Key informants were purposefully sampled based on their involvement with perinatal audit and included two regional PPIP coordinators, sub-district health managers, and clinical staff. Interviews were conducted with at least 10 staff per case or until saturation had been reached,

with the exception of case D where only five staff were available. In total, 56 key informants were included. Table S1.1 provides details about key informants and meetings observed.

Data collection

Data collection tools included a key informant interview guide and a meeting observation guide. Fieldwork and data collection took place from October 2019 to March 2020 ranging from half of a day to five days per site. MK conducted the fieldwork and sent a summary report of preliminary findings and reflection to the research team within one week of visiting the site. Key informant interviews were in English and ranged from 20 minutes to one hour. All interviews were conducted individually with the exception of case D, which were done in two groups. Non-participant observations occurred at seven meetings: two provincial PPIP meeting, three sub-district perinatal review meetings (M&M meetings), one monitoring and evaluation (M&E) meeting, and one other staff meeting.

For the standard implementation assessments, data collection included: 1) administration of a standardised, semistructured questionnaire to facility health workers supporting MPDSR-related activities who were present on the day of the visit, and 2) observations of perinatal audit-related documents and activities in the facility (e.g., review of perinatal review meeting notes). The questionnaire was completed with one of the identified PPIP focal points and verified with other stakeholders as needed. A tool developed to measure the stage of MPDSR implementation was applied.[6]

Data management and analysis

Interviews were recorded and transcribed. Transcripts, observation and reflection notes were compiled and analysed using Atlas.ti (v9) by MK with oversight from AG. Thematic analysis was used applying an analysis framework derived from the extended Normalization Process Theory,[7] an implementation theory used to consider broader social systems in which interventions are implemented. Using thematic analysis and an iterative process, the emerging themes were considered in relation to the analysis framework to inform the findings of the research. A report was developed for each case study by MK and received inputs from all authors.

To derive a cumulative implementation progress score for each facility, the quantitative data were analysed using an implementation progress monitoring tool.[6] An implementation progress score was calculated for each sub-district across six stages of implementation, with each stage having a weighted score based on specific points. For each stage, MK considered all relevant collected data to assign stage-specific points, contributing to a possible total score of 30. Data from the facility and subnational key informant questionnaires were extracted into a database to tabulate descriptive means and frequencies of explanatory variables and progress markers. Qualitative data were analysed using thematic content analysis. To determine the leading facility-reported barriers and enablers to MPDSR implementation, the team analysed the frequency of qualitative responses from facility interviews based on the thematic content analysis and considered the frequency of relevant progress markers.

Rigour and ethics

Measures were taken to ensure rigour of the case-study approach,[3,8] such as engagement with stakeholders prior to data collection; voluntary participation of participants; seeking peer and expert feedback; audit trail with clear mapping of the research process; and triangulation of data sources. A short report was provided to sub-district representatives to verify results with

the stakeholders. Permission to take photographs of documents and training materials was given from sub-district health administrators, with commitment to not to include sensitive information and identifiers.

The ethics approval from the Higher Degrees Committee of the University if the Western Cape was given on 09 November 2018, and approval was received from the Provincial Department of Health in July 2019 (NHRD Number: WC_201906_006). Authorization to conduct the study was granted from the Department of Health of the Western Cape Province.

Supplement to: Kinney MV, George AS, Rhoda N, Pattinson RC, Bergh AM. From pre-implementation to institutionalization: lessons from sustaining a perinatal audit program in South Africa. *Glob Health Sci Pract*. 2023;11(1):e2200213. https://doi.org/10.9745/GHSP-D-22-00213

Table S2 Distribution of participants and meeting observations

		District/Regional	Case A	Case B	Case C	Case D	Case E	Total
	TOTAL MEETINGS	2	1	2	3	0	2	10
Non-participant observation of meetings	PPIP provincial meeting	2						2
	M&M meetings		1	2	1		1	3
	M&E meetings				1		1	1
	Other meetings				1			1
	TOTAL INTERVIEWS	9	10	11	10	5	8	56
National, provincial, regional and district level	National actors*	3						3
	Provincial actors**	3						3
	Regional and district actors	6						6
Sub-district level	Medical manager		1	1	n/a	1	n/a	3
	Nursing manager		1	1	1	1	1	5
	Clinical manger		1	n/a	1	0	0	2
	Information manager		1	1	0	1	n/a	3
	Quality assurance manager		n/a	0	1	0	0	1
	PHC manager		0	0	1	0	0	1
	comprehensive service manager		0	0	0	0	n/a	0
	Pharmacy supervisor		0	0	0	0	1	1
	Information officer		0	0	1	1	1	3
Facility level	Family physician		1	1	1	0	n/a	3
	Medical officer (incl Senior and Registrar)		0	2	2	0	2	6
	Operational manager (facility)		n/a	1	n/a	n/a	n/a	1
	Operational manager (maternity)		1	n/a	1	1	n/a	3
	Professional Nurse		2	2	1	0	1	6
	Enrolled nurse		0	0	0	0	1	1
PHC level	PHC clinic manager		1	1	0	0	1	3
	PHC nurse practitioner		1	1	0	0	0	2

Note: n/a indicates that this position did not exist at this site or was not occupied at the time of the data collection.
Key: M&E, monitoring and evaluation; M&M, morbidity and mortality; PPIP, Perinatal Problem Identification Program; PHC, primary health care
* Included one academic, one former National Department of Health (DOH) employee, one District Clinical Specialist Paediatrician from another province
** Included one WC DOH employee, one academic, one retired WC DOH

Supplement to: Kinney MV, George AS, Rhoda N, Pattinson RC, Bergh AM. From pre-implementation to institutionalization: lessons from sustaining a perinatal audit program in South Africa. *Glob Health Sci Pract*. 2023;11(1):e2200213. https://doi.org/10.9745/GHSP-D-22-00213

SUPPLEMENT 3. DESK REVIEW ADDITIONAL INFORMATION

Table S3.1 Documents identified in the desk review

Type of document	Documents
Saving Babies Reports	Saving Babies Report 2000 (Report 1)<br>Saving Babies Report 2001 (Report 2)<br>Saving Babies Report 2002 (Report 3)<br>Saving Babies Report 2003 (Report 4)<br>Saving Babies Report 2003-2005 (Report 5)<br>Saving Babies Report 2006-2007 (Report 6)<br>Saving Babies Report 2008-2009 (Report 7)<br>Saving Babies Report 2010-2011 (Report 8)<br>Saving Babies Report 2012-2013 (Report 9)
NaPeMMCo reports	National Perinatal Mortality and Morbidity Committee (NaPeMMCo) Triennial Report (2008-2010): National Perinatal Mortality and Morbidity Committee 2011.<br><br>National Perinatal Mortality and Morbidity Committee (NaPeMMCo) Triennial Report (2010-2013). Short Report 2014: National Perinatal Mortality and Morbidity Committee 2014.<br><br>Saving Babies 2014-2016: Triennial report on perinatal mortality in South Africa Pretoria: South Africa National Department of Health; 2016<br><br>National Perinatal Mortality and Morbidity Committee (NaPeMMCo) Triennial Report (2017-2019): National Perinatal Mortality and Morbidity Committee 2021.
National policies and guidelines	South African Government. Births and Deaths Registration Act 51 of 1992 Pretoria: Government Gazette; 1992 [Gazette 14182]<br><br>National Department of Health. Strategic Plan (2012-2016) for Maternal, Newborn Child and Women's Health (MNCWH) and Nutrition. Pretoria: National Department of Health, 2012.<br><br>National Department of Health. Guidelines for maternity care in South Africa: 4th edition Pretoria: South African National Department of Health; 2016<br><br>National Department of Health. The Ideal Hospital Realisation and Maintenance Framework Manual Pretoria: South African National Department of Health; 2018<br><br>National Department of Health. South African maternal, perinatal and neonatal health policy Pretoria: South African National Department of Health; 2021

**Type of document**	**Documents**
Perinatal Priorities Conference proceedings	Priorities in Perinatal Care Association of South Africa. Proceedings Database Pretoria: University of Pretoria; 2022. Available from: https://www.perinatalpriorities.co.za/proceedings-database/
Educational materials	Woods DL, Pattinson RC, Greenfield D, et al. Saving Mothers and Babies: Assessing and reducing mortality rates in your hospital In: Woods DL, ed. Perinatal Education Program online courses: Bettercare Electronic Book Works 2008.
Other reports	Bergh AM, Pattinson R, Belizan M, et al. Completing the audit cycle for quality care in perinatal, newborn and child health Pretoria: 2011
Academic articles	Allanson ER, Pattinson RC. Quality-of-care audits and perinatal mortality in South Africa. Bull World Health Organ 2015;93(6):424-8. doi: 10.2471/BLT.14.144683<br><br>Belizan M, Bergh AM, Cilliers C, et al. Stages of change: A qualitative study on the implementation of a perinatal audit program in South Africa. BMC Health Serv Res 2011;11:243. doi: 10.1186/1472-6963-11-243<br><br>Swartz A, LeFevre AE, Perera S, et al. Multiple pathways to scaling up and sustainability: an exploration of digital health solutions in South Africa. Globalization and health 2021;17(1):77. doi: 10.1186/s12992-021-00716-1 [published Online First: 2021/07/08]<br><br>Mukinda FK, George A, Van Belle S, et al. Practice of death surveillance and response for maternal, newborn and child health: a framework and application to a South African health district. BMJ Open 2021;11(5):e043783. doi: 10.1136/bmjopen-2020-043783<br><br>Rhoda NR, Greenfield D, Muller M, et al. Experiences with perinatal death reviews in South Africa--the Perinatal Problem Identification Program: scaling up from program to province to country. BJOG 2014;121 Suppl 4:160-6. doi: 10.1111/1471-0528.12997<br><br>Rhoda N, Velaphi S, Gebhardt GS, et al. Reducing neonatal deaths in South Africa: Progress and challenges. *South African Medical Journal* 2018;108:s9. doi: 10.7196/SAMJ.2017.v108i3b.12804<br><br>Woods DL. Improving neonatal care in district and community health facilities in South Africa. Paediatr Int Child Health 2015;35(3):187-91. doi: 10.1179/2046905515Y.0000000031<br><br>Woods DL, Pattinson RC, Greenfield D, et al. Saving Mothers and Babies: Assessing and reducing mortality rates in your hospital In: Woods DL, ed. Perinatal Education Program online courses: Bettercare Electronic Book Works 2008.

Table S3.2 Timeline of the perinatal audit program milestones

Year	Activity
1982	Priorities in Perinatal Care Association of South Africa established and begins annual conference.
1992	The registration of perinatal deaths in South Africa falls under the mandate of the Department of Home Affairs (DHA) and is governed by the Births and Deaths Registration Act 1992 (Act No. 51 of 1992) (Republic of South Africa, 1992). This Act was last amended in 2010 as the Births and Deaths Registration Amendment Act (Act No.18 of 2010), with the regulations of the Act amended in 2014 (Republic of South Africa, 2010; Republic of South Africa, 2014). According to the principal Act, 'A stillborn in relation to a child, means that it has at least 26 weeks of intra-uterine existence but showed no sign of life after complete birth (Republic of South Africa, 1992:5). In terms of the Regulations on the Registration of Births and Deaths, stillbirths and deaths have to be registered within 72 hours of occurrence (Republic of South Africa, 2014). While the certification of the occurrence of a stillbirth and provision of information on causes of death may be done by a professional nurse, all other deaths, including neonatal deaths, have to be certified by a medical practitioner. However, if any death was not due to natural causes, the medical practitioner is obliged to report such a death to a police officer. Subject to the Inquests Act, 1959 (Act No.58 of 1959), the police officer shall investigate the circumstances of the death while a forensic pathologist should perform a medico-legal post mortem to determine the causes of death.
>1992	Dr. Pattinson moved to Kalafong Hospital and began to collect data on perinatal death to identify avoidable factors. (Paper published SAMJ 1994)
1993	Paper based system developed by Dr. Pattinson (interview)
1994	Software developed and designed by Dr Johan Coetzee – a registrar (interview). Development of the program started in 1991 when Johan developed the simple DOS program and this was piloted in 1994 (Saving Babies 2008)<br>• First page: Number of births, deaths and other data (syphilis, teenage pregnancy) per weight classification and time of death<br>• Second page: Allocation of cause<br>• Third page: Allocation of avoidable factors - ICA Solution Audit System (Identification, Cause, Avoidable Factors, Solutions)<br><br>Priorities in Perinatal Care Association of South Africa formalize with a constitution.
1996	Field testing of PPIP – a number of reports produced (Mpumalanga: Witbank, Middleburg) (interview) (Priorities 1997 conference abstracts)
1998-1999	PPIP program change to windows and became operationalized (interview)<br><br>The national PPIP database administered by the MRC Maternal and Infant Health Care Strategies Research Unit was set up on 1 October 1999 (Saving Babies 2003-5)

**Year**	**Activity**
1995	In 1995, South Africa ratified the United Nations Convention on the Rights of the Child (UNCRC).
2000	Published: *Saving Babies: A perinatal care survey of South Africa*<br><br>Perinatal Care Survey of South Africa Workshop - 12-14 November 2000 (Saving Babies, 2000)
2001	Published: *Saving Babies 2001: Second perinatal care survey of South Africa*<br><br>Perinatal Care Survey of South Africa Workshop 26th – 28th November 2001 (Saving Babies, 2001)
2002	Published: *Saving Babies 2003: Third Perinatal Care Survey of South Africa.*<br><br>Perinatal Care Survey of South Africa Workshop 18-20 November 2002 (Saving Babies, 2002)
2003	Published: *Saving Babies 2003: Fourth Perinatal Care Survey of South Africa.*<br><br>The Perinatal Education Program has produced a training manual using maternal and perinatal audits called Manual 5: Saving Mothers and Babies. It is a very valuable tool for sites wanting to start with audits or to introduce new members of staff to maternal and perinatal audits
2004	Release of PPIP v2
2005	Published: *Saving Babies 2003-2005: Fifth Perinatal Care Survey of South Africa*
2008	NaPemCo established<br>*National Perinatal and Neonatal Morbidity and Mortality Committee(NaPeMMCo)*<br>The National Perinatal and Neonatal Morbidity and Mortality Committee was established in March 2008 by the Minister of Health. The purpose of NaPeMMCo was to audit all perinatal and neonatal deaths occurring in the country and to produce annual reports. The committee was also set up to make recommendations on solutions for the reduction of perinatal and neonatal deaths.<br><br>DHIS captures most births - PPIP begins to use DHIS as source for denominator (interview)
2009	A workshop was held from 15-16 September 2009 in which 48 participants from all nine provinces in South Africa deliberated the audit cycle and its completion.<br><br>Published: *Saving Babies 2006-2007: Sixth Perinatal Care Survey of South Africa*
2011	Published: *Saving Babies 2008-2009: Seventh Perinatal Care Survey of South Africa*

Year	Activity
	Published: *National Perinatal Morbidity and Mortality Committee: Triennial Report (2008-2010)*<br><br>Published: Bergh AM, Pattinson R, Belizan M, et al. Completing the Audit Cycle for Quality Care in Perinatal, Newborn and Child Health. Pretoria: MRC Research Unit for Maternal and Infant Health Care Strategies, Univeristy of Pretoria; 2011.<br><br>Published: Belizán M, Bergh AM, Cilliers C, et al. Stages of Change: A Qualitative Study on the Implementation of a Perinatal Audit Program in South Africa. BMC Health Serv Res. 2011;11:243. doi:10.1186/1472-6963-11-243
2012	A long and healthy life for all South Africans' is one of the key development objectives outlined in the National Development Plan (NDP) adopted by the South African government in 2012 (National Planning Commission, 2012). In order to reach the NDP goal, the national DoH advocates for the reduction of neonatal and child mortality rates, including improvement of health care systems (DoH, 2014)<br><br>In 2012, the Campaign for Accelerated Reduction in Maternal and Child Mortality in Africa (CARMMA) was launched in South Africa.<br>The Maternal, Neonatal, Child and Women's Health and Nutrition (MNCWH&N) Strategic Plan 2012–2016 aims to identify and strengthen priority interventions that would have the greatest impact on reducing maternal, neonatal and child mortality. Included objective: Institutionalize reviews of maternal, perinatal, neonatal and child deaths. Monthly review meetings are required; strong recommendation for facilities to use PPIP.<br><br>In 2012, National Department of Health recommitted to achieving the Millennium Development Goal 4 targeted towards reducing under-five child mortality, including perinatal deaths. As a result, the PPIP became mandatory for all facilities (hospitals and 24-hour clinics) rendering a maternity services and caring for newborns (Pattinson & Rhoda, 2014:26)<br><br>Published: *National Perinatal Morbidity and Mortality Committee: Interim Report (2010-2011)*
2013	PPIP v3 released<br><br>Published: *Saving Babies 2010-2011: Eighth Perinatal Care Survey of South Africa*<br><br>Establishment of the National Neonatal Co-ordinating Committee (NNCC), which provided a forum within the NDoH to co-ordinate and give oversight to improvements in newborn care in SA. (Rhoda et al SAMJ)
2014	Published: *Saving Babies 2012-2013: Ninth Perinatal Care Survey of South Africa*<br>Published: *National Perinatal Morbidity and Mortality Committee: Triennial Report (2010-2013)*

**Year**	**Activity**
2015	Mid-term review of the MNCWH&N Strategic Plan does not report on reviews of perinatal deaths; includes CHIP in recommendations (not PPIP or MAMA)
2016	National Department of Health. Guidelines for maternity care in South Africa: 4th edition includes PPIP and instructions for how to conduct perinatal death review meetings.<br><br>Published: *Saving Babies 2014-2016: Triennial Report on perinatal mortality in South Africa*
2018	"Plans are afoot by the NDoH to synchronise and align PPIP and Child PIP with the DHIS" Rhoda et al 2018 SAMJ
2018	The Ideal Hospital initiative started and includes monthly M&M meetings as requirement.
2020	Completed but not published: *National Perinatal Morbidity and Mortality Committee: Triennial Report (2017-2019)*
2021	The 2021 Maternal, Perinatal and Neonatal Health (MPNH) Policy sets an objective to develop a sustainable surveillance system for maternal, perinatal and neonatal morbidity and mortality and argues that "Data governance for MPNH delivery in South Africa is fragmented, with multiple data sources for maternal and neonatal mortality, at times with divergent views." [9] The policy does not mention PPIP specifically. Additionally, the section on MPDSR only includes the surveillance component of the cycle, does not specify which system should be used for notification, and does not link to perinatal review meetings. Clinical audits and related review meetings are presented in the section on quality of care.

Supplement to: Kinney MV, George AS, Rhoda N, Pattinson RC, Bergh AM. From pre-implementation to institutionalization: lessons from sustaining a perinatal audit program in South Africa. *Glob Health Sci Pract*. 2023;11(1):e2200213. https://doi.org/10.9745/GHSP-D-22-00213

Figure S3. Initiatives to improve neonatal care

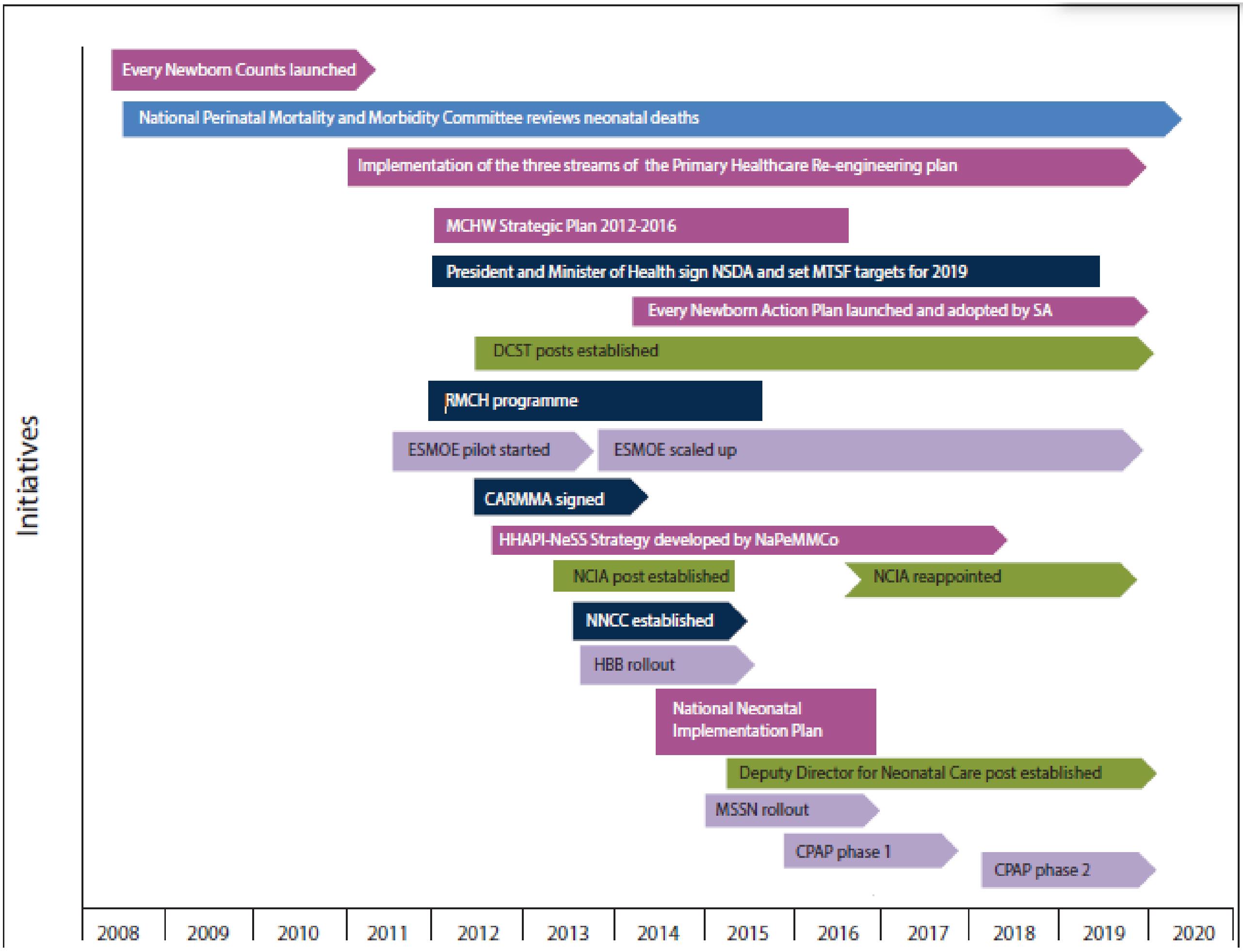

Source: Reprinted from Rhoda et al SAMJ 2018[4]

Key: MCHW = Maternal, Newborn, Child and Women's Health and Nutrition in South Africa 2012 - 2016; NDSA = negotiated service delivery agreement; MTSF= medium-term strategic framework; SA = South Africa; DCST = district clinical specialist team; RMCH = Reproductive, Maternal and Child Health; ESMOE = Essential Steps in the Management of Obstetric Emergencies; CARMMA = Campaign for the Accelerated Reduction of Maternal Mortality in Africa; NCIA - neonatal care improvement advisor; NNCC = National Neonatal Co-ordinating Committee; HBB = Helping Babies Breathe; MSSN = managing small and sick newborns; CPAP = continuous positive airway pressure.

Supplement to: Kinney MV, George AS, Rhoda N, Pattinson RC, Bergh AM. From pre-implementation to institutionalization: lessons from sustaining a perinatal audit program in South Africa. *Glob Health Sci Pract*. 2023;11(1):e2200213. https://doi.org/10.9745/GHSP-D-22-00213

SUPPLEMENT 4. POLICY ANALYSIS

Phase 1 (1992-2007): The start of the perinatal audit program (pre-implementation)

- *Context*: District Health Information Software (DHIS) introduced and scaled nationally to collect routine data; multiple maternal and newborn health (MNH) programs initiated; PPIP expands from 27 facilities in 2000 to 244 facilities.
- *Content*: Policy to register perinatal deaths (1992); Convention on the Rights of the Child signed (1993); Millennium Development Goal commitment (2000).
- *Actors*: Bottom up approach from committed champions who initiated PPIP and led the roll out; Saving Babies Technical Task Team established; University of Pretoria established Maternal and Infant Health Care Strategies Research Unit (1997) (an extra-mural unit of the South African Medical Research Council).
- *Process*: User friendly software developed and filled a gap in the routine data systems; annual Perinatal Priorities Conferences with learning shared; multiple PPIP workshops along with regularly published Saving Babies reports; Perinatal Education Program (PEP) in South Africa established (1993) to provide continued learning opportunities for clinical staff.[10]

Phase 2 (2008-2012): The scale up of the perinatal audit program (implementation)

- *Context:* Implementation of MNH programs in response to PPIP findings; PPIP expands from 275 facilities to 588 facilities.[11]
- *Content:* The MNCWH&N Strategic Plan 2012–2016 includes indicator making perinatal death reviews mandatory in hospitals; District Clinical Specialist Teams established.
- *Actors*: Formalization of network with ongoing engagement from original champions and expansion of network through establishment of National Perinatal Committee with regular reporting of perinatal mortality to the Minister of Health.
- *Process*: One workshop along with biannual Saving Babies reports; annual Perinatal Priorities Conferences with learning shared; implementation research;[4,5,12] provincial PPIP trainings; PEP develops *Saving Mothers and Babies* curriculum (2008), which includes perinatal death audit.[10,13]

Phase 3 (2013-2019): The sustaining of the perinatal audit program (institutionalization)

- *Context*: Implementation of MNH programs in response to PPIP findings (e.g. Helping Babies Breathe, management of small and sick newborns); over 75% of births recorded through PPIP.[14]
- *Content*: Sustainable Development Goal Commitment; Adaptation of Every Newborn Action Plan; 2016 Maternity Guidelines include use of PPIP and provides a "how to guide" for conducting perinatal death review; 2021 Maternal, Perinatal, and Newborn Health Policy does not include PPIP.

- *Actors*: Institutionalization of actors and networks to oversee implementation including new posts in National Department of Health (e.g. neonatal care improvement advisor and Deputy Director for Neonatal Care post) and establishment of National Neonatal Coordinating Committee; University of Pretoria Research Centre for Maternal, Fetal, Newborn and Child Health Care Strategies established taking over role of training and technical support (2016).
- *Process*: Reporting and oversight embedded in system demonstrated by biannual Saving Babies report as well as triennial National Perinatal Mortality and Morbidity Committee report; annual Perinatal Priorities Conferences with learning shared.

A timeline of activities across the four components of the health policy analysis triangle were adapted from: Kinney, MK. (2019, September 17-19). 25 years of the Perinatal Problem Identification Program: Unpacking mortality reduction linked to factors that influenced the development and implementation process. [Conference oral presentation]. PHASA 2019 Conference, Athlone, Cape Town.

Supplement to: Kinney MV, George AS, Rhoda N, Pattinson RC, Bergh AM. From pre-implementation to institutionalization: lessons from sustaining a perinatal audit program in South Africa. *Glob Health Sci Pract*. 2023;11(1):e2200213. https://doi.org/10.9745/GHSP-D-22-00213

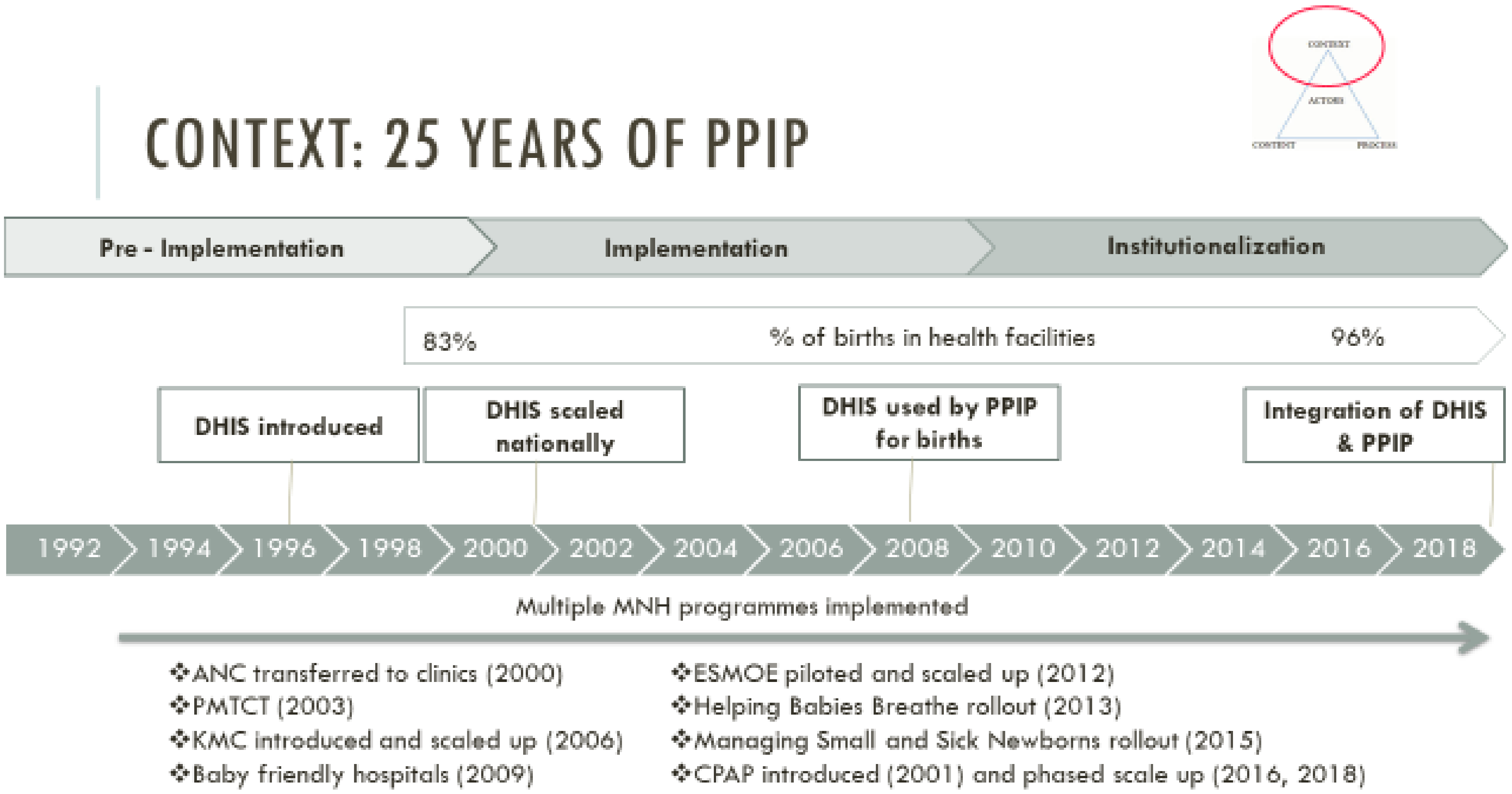

Supplement to: Kinney MV, George AS, Rhoda N, Pattinson RC, Bergh AM. From pre-implementation to institutionalization: lessons from sustaining a perinatal audit program in South Africa. *Glob Health Sci Pract*. 2023;11(1):e2200213. https://doi.org/10.9745/GHSP-D-22-00213

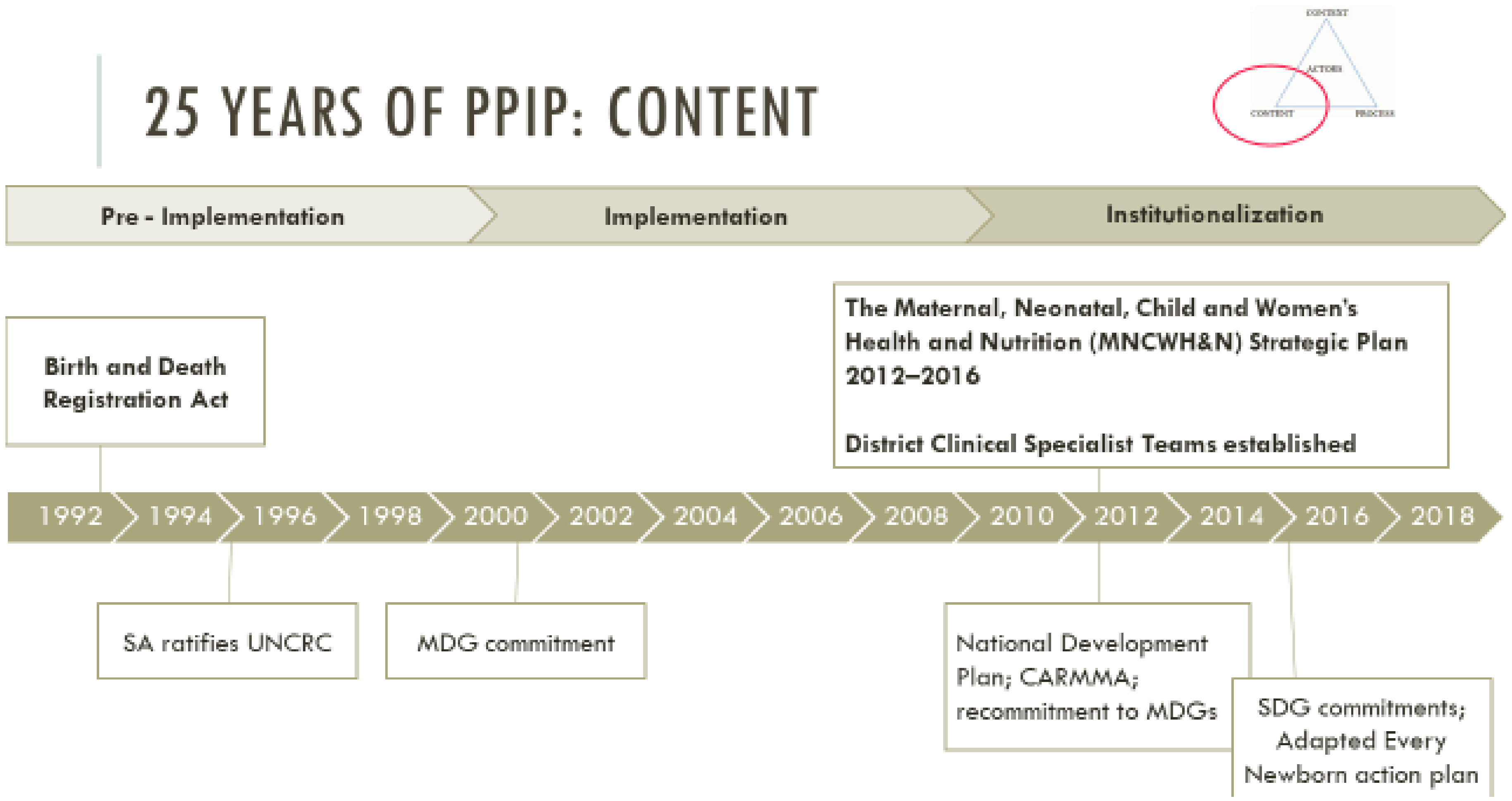

Supplement to: Kinney MV, George AS, Rhoda N, Pattinson RC, Bergh AM. From pre-implementation to institutionalization: lessons from sustaining a perinatal audit program in South Africa. *Glob Health Sci Pract*. 2023;11(1):e2200213. https://doi.org/10.9745/GHSP-D-22-00213

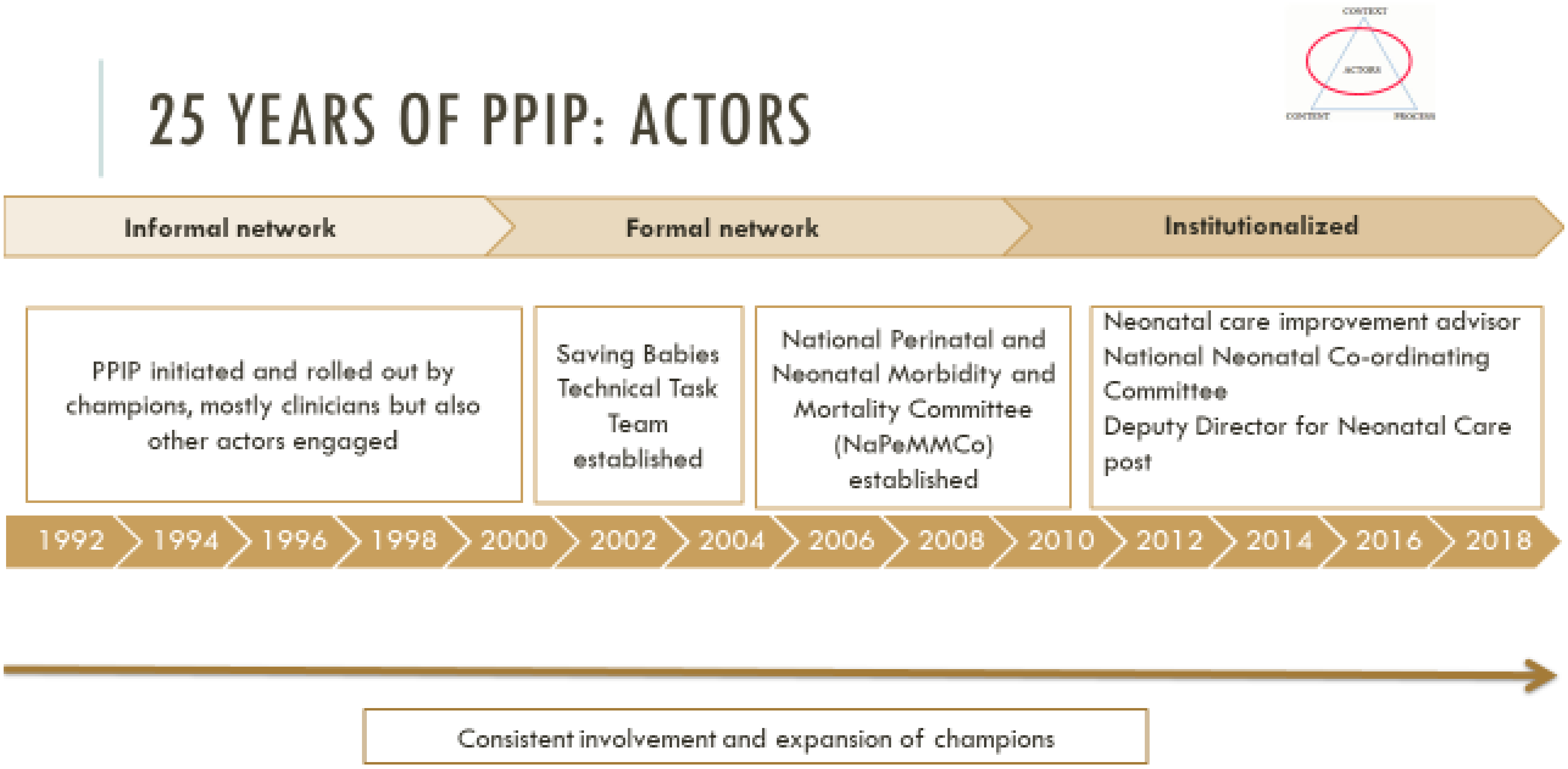

Supplement to: Kinney MV, George AS, Rhoda N, Pattinson RC, Bergh AM. From pre-implementation to institutionalization: lessons from sustaining a perinatal audit program in South Africa. *Glob Health Sci Pract*. 2023;11(1):e2200213. https://doi.org/10.9745/GHSP-D-22-00213

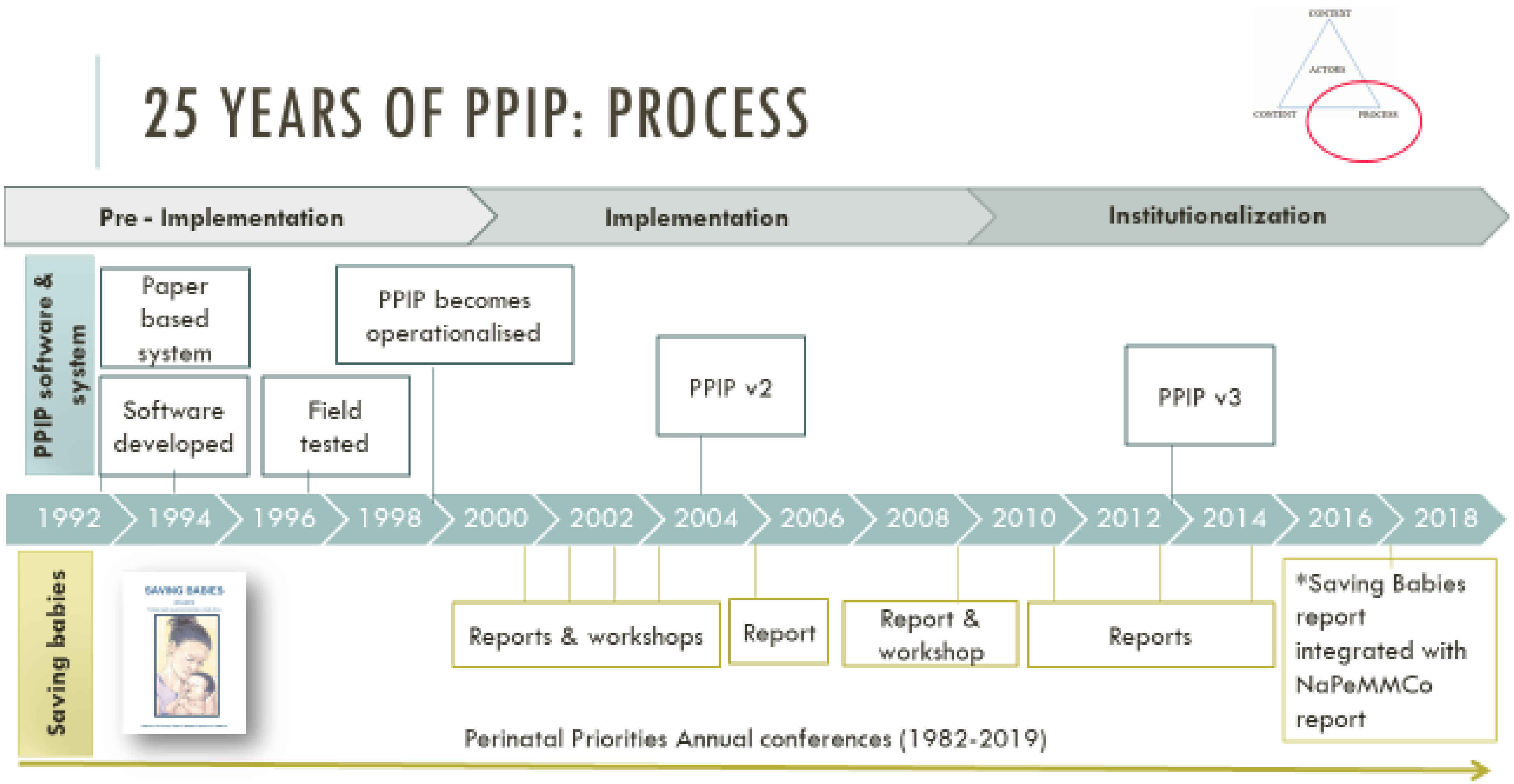

Supplement to: Kinney MV, George AS, Rhoda N, Pattinson RC, Bergh AM. From pre-implementation to institutionalization: lessons from sustaining a perinatal audit program in South Africa. *Glob Health Sci Pract*. 2023;11(1):e2200213. https://doi.org/10.9745/GHSP-D-22-00213

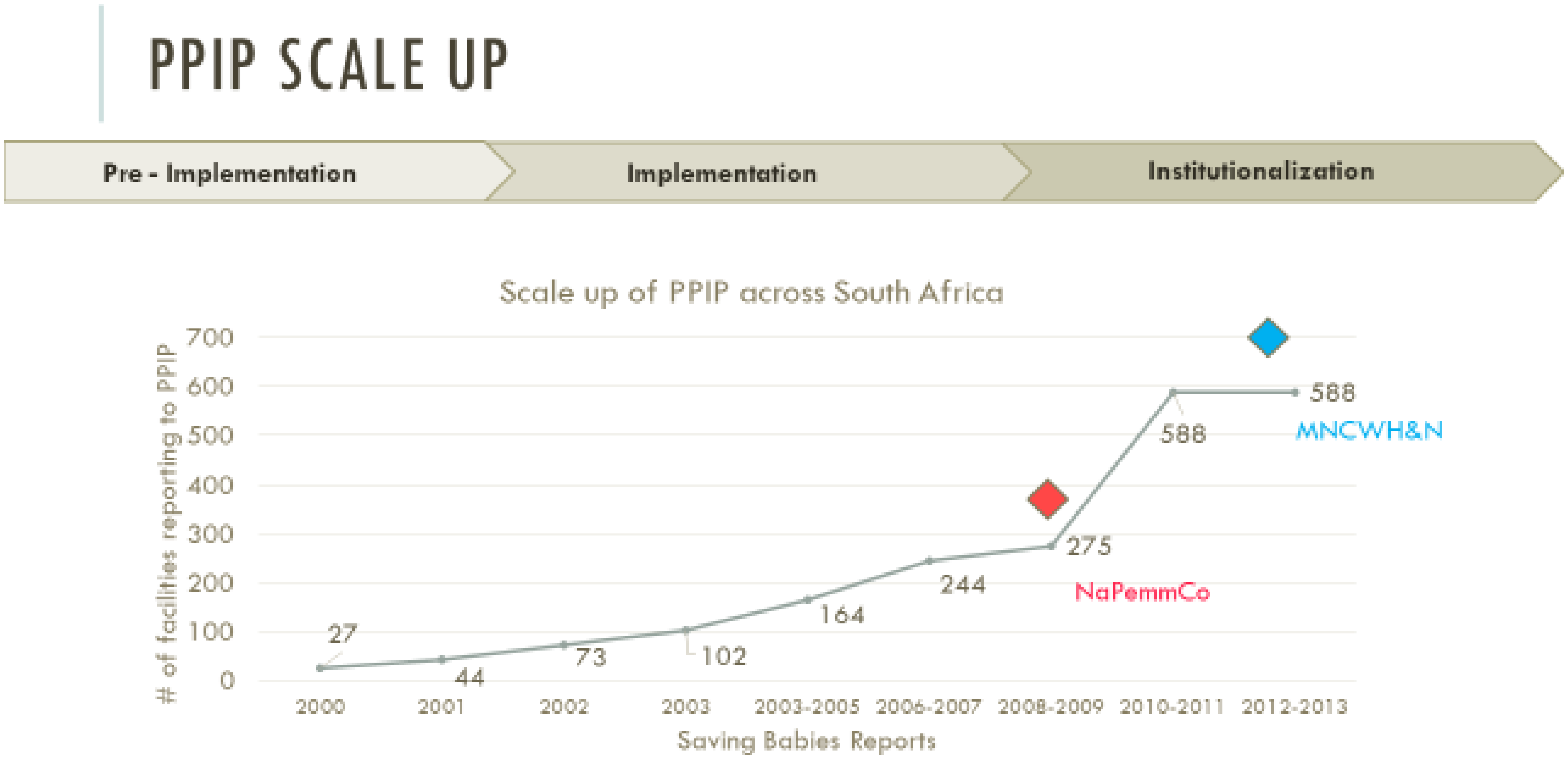

Supplement to: Kinney MV, George AS, Rhoda N, Pattinson RC, Bergh AM. From pre-implementation to institutionalization: lessons from sustaining a perinatal audit program in South Africa. *Glob Health Sci Pract*. 2023;11(1):e2200213. https://doi.org/10.9745/GHSP-D-22-00213

SUPPLEMENT 5. PROVINCIAL AND SUBNATIONAL STRUCTURES

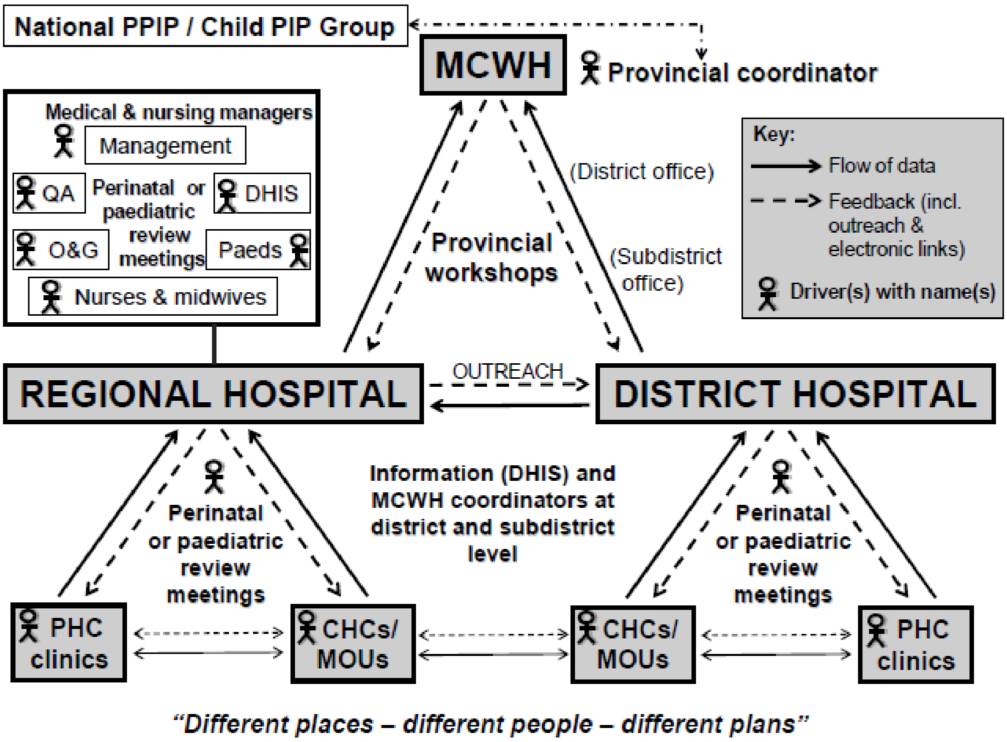

PPIP, Perinatal Problem Identification Program; Child PIP, Child Problem Identification Program; MCWH, Maternal, Child and Women's Health; QA, Quality Assurance; DHIS, District Health Information Software; O&G, Obstetrics and Gynaecology; Paeds, Paediatrics; PHC, Primary health care; CHC, Community health centre; MOU, Midwife-led obstetric unit
Source: Image used with permission from Bergh et al (2011) Completing the audit cycle for quality care in perinatal, newborn and child health[12]

Supplement to: Kinney MV, George AS, Rhoda N, Pattinson RC, Bergh AM. From pre-implementation to institutionalization: lessons from sustaining a perinatal audit program in South Africa. *Glob Health Sci Pract*. 2023;11(1):e2200213. https://doi.org/10.9745/GHSP-D-22-00213

SUPPLEMENT 6. CASE STUDY RESEARCH ADDITIONAL INFORMATION

Table S6.1: Individuals responsible for activities relating to perinatal audit

		Case study A	Case study B	Case study C	Case study D	Case study E
**Identification and reporting**	Capturing data in PPIP software	Operational manager of maternity ward	Operational manager of hospital	Information officer, operational manager of maternity ward	Information officer, operational manager of maternity ward	Information officer. matron
	Ensuring death notification form completed	Matron	Operational manager of hospital	Information officer	Information officer	Information officer
**Reviewing deaths**	Logistics	Clinical manager	Family physician	Family physician, QA manager	Clinical manager	Matron
	Case and data preparation	Medical officer (on duty that month), operational manager of maternity ward	Medical officers, operational manager of hospital	Family physician, Operational manager of maternity ward, QA manager, information officer	Medical officer (responsible for case), operational manager of maternity ward, information officer	Medical officer
	Facilitation	Regional PPIP coordinator	Regional PPIP coordinator	Family physician, regional PPIP coordinator	Clinical manager, regional PPIP coordinator	Medical officer
	Record minutes	Operational manager of maternity ward	Operational manager of maternity ward or matron	QA manager	QA manager	Matron

		**Case study A**	**Case study B**	**Case study C**	**Case study D**	**Case study E**
**Analysis and recommendations**	PPIP data	Regional PPIP coordinator	Regional PPIP coordinator	Information officer	Information officer	Information manager (sub-district)
	Case reviews	Regional PPIP coordinator in discussion with staff at M&M meeting	Regional PPIP coordinator in discussion with staff at M&M meeting	Family physician, regional PPIP coordinator in discussion with staff at M&M meeting	Clinical manager in discussion with staff at M&M meeting	Medical officer
**Response and actions**	Responsible actions are done	Sub-district managers, regional PPIP coordinator	Sub-district managers (matron, medical manager), family physician, operational manager of hospital, regional PPIP coordinator	Sub-district managers, operational manager of maternity ward maternity, regional PPIP coordinator	Sub-district managers, clinical manager, operational manager of maternity ward maternity, regional PPIP coordinator	Matron, sub-district managers
	Responsible for checking actions are done	No formal reporting system	No formal reporting system	QA manager	Clinical manager/QA manager	Matron, sub-district managers
	**Different actors identified as the perinatal audit focal person by colleagues interviewed**	Matron, operational manager of maternity ward, clinical Manager	Family physician, operational manager of hospital, medical officer	Family physician, QA manager	Operational manager of maternity ward, information officer, clinical manager	Matron

QA, quality assurance; PPIP, Perinatal Problem identification Program; M&M, morbidity and mortality

Supplement to: Kinney MV, George AS, Rhoda N, Pattinson RC, Bergh AM. From pre-implementation to institutionalization: lessons from sustaining a perinatal audit program in South Africa. *Glob Health Sci Pract*. 2023;11(1):e2200213. https://doi.org/10.9745/GHSP-D-22-00213

Figure S6.1 Demonstration of implementation gaps through photos of M&M meeting minutes (Region 1: Case studies A and B)

Case study A

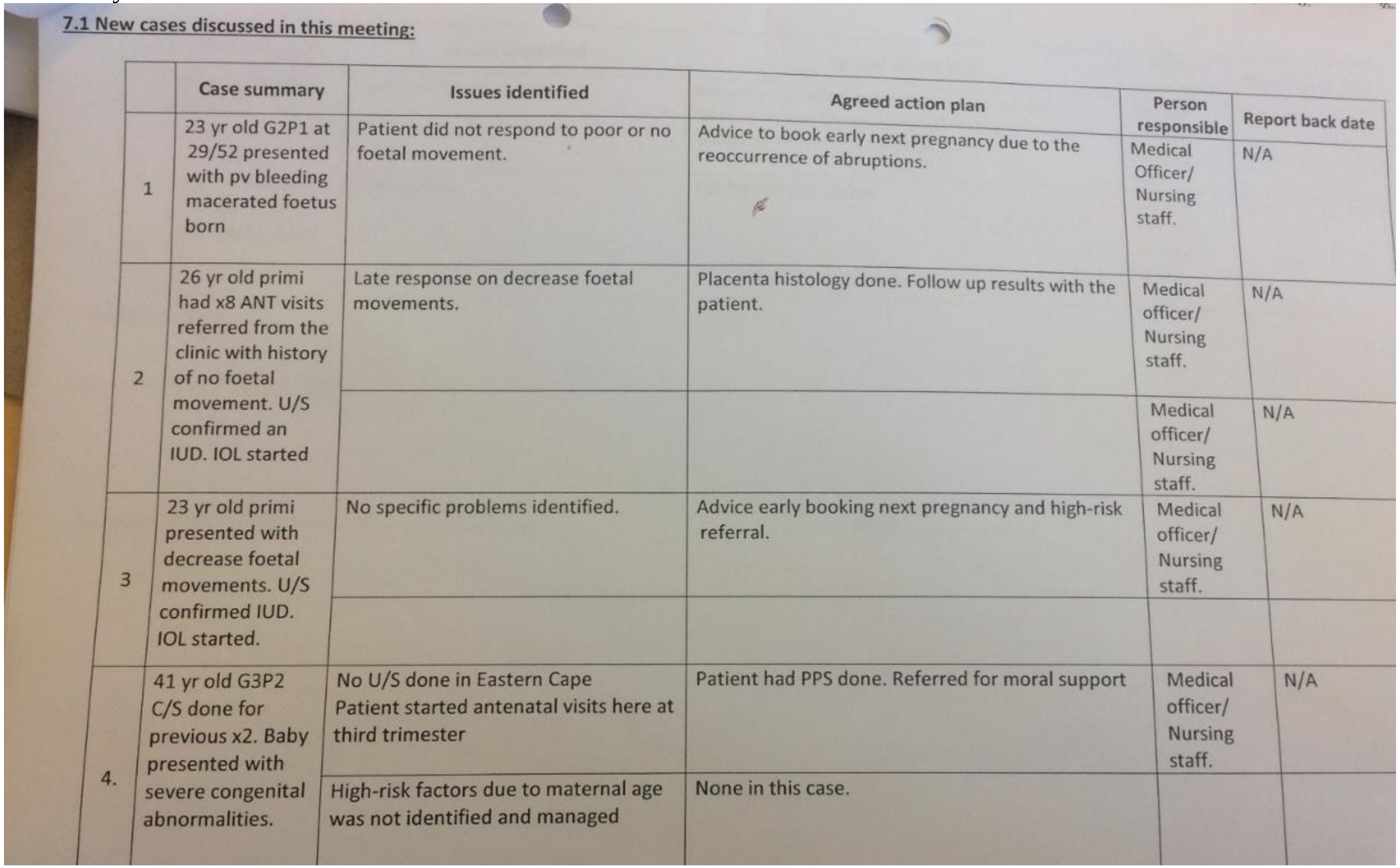

7.1 New cases discussed in this meeting:

	Case summary	Issues identified	Agreed action plan	Person responsible	Report back date
1	23 yr old G2P1 at 29/52 presented with pv bleeding macerated foetus born	Patient did not respond to poor or no foetal movement.	Advice to book early next pregnancy due to the reoccurrence of abruptions.	Medical Officer/ Nursing staff.	N/A
2	26 yr old primi had x8 ANT visits referred from the clinic with history of no foetal movement. U/S confirmed an IUD. IOL started	Late response on decrease foetal movements.	Placenta histology done. Follow up results with the patient.	Medical officer/ Nursing staff.	N/A
				Medical officer/ Nursing staff.	N/A
3	23 yr old primi presented with decrease foetal movements. U/S confirmed IUD. IOL started.	No specific problems identified.	Advice early booking next pregnancy and high-risk referral.	Medical officer/ Nursing staff.	N/A
4.	41 yr old G3P2 C/S done for previous x2. Baby presented with severe congenital abnormalities.	No U/S done in Eastern Cape Patient started antenatal visits here at third trimester	Patient had PPS done. Referred for moral support	Medical officer/ Nursing staff.	N/A
		High-risk factors due to maternal age was not identified and managed	None in this case.		

Supplement to: Kinney MV, George AS, Rhoda N, Pattinson RC, Bergh AM. From pre-implementation to institutionalization: lessons from sustaining a perinatal audit program in South Africa. *Glob Health Sci Pract*. 2023;11(1):e2200213. https://doi.org/10.9745/GHSP-D-22-00213

Case study B

7.1 New cases discussed in this meeting:

	Case summary	Issues identified	Agreed action plan	Person responsible	Report back date
1	Case 1, G3, P2<br>37 weeks gestation.<br>Urine: Prot +++, Glucose +++, could be PET.<br>Cause of death: abruptio with Hypertension. BP later 140/102, late onset PET	In labour, 3:20 Abruption suspected. 1 cm dilation, bradycardia, FH 60/70bpm<br>CTG heartrate clearly not the mother. Flat, no variability. Tracing to get baby out alive is slim.	Abruption with dead baby, (DIC / end up with C/S) Could transfer to WPH – access to Lab to exclude HELLP.	Midwife, doctor	N/A
		Before theatre: bleeding times: Theatre, bloods given.	If baby still alive, don't worry with bleeding times/ clot factors. It delays the surgery.	Surgeon	N/A
		Spinal done – severe fetal distress	Spinal not recommended. On edge of not keeping BP going, vasodilation, go for GA.	Anaesthetist	N/A
2	[redacted]<br>Fresh S/B IUD (involute / re-absorbed twin after 24 wks).<br>2nd twin alive.<br>Booked X1.<br>1 visit @10 weeks, then 1 August 2019.	Despite fact that she had 2 previous C/s, normal to deliver normal.<br>Risk of Uterine rupture higher. Record risk history on Antenatal booking	Patient high risk: fill in HIV+ write CD4 & Viral load. PN at night don't know the blood type / HIV & RPR status. Even if somebody on ART's, write the last dose.	PHC staff at Clinic	N/A
		2 previous M/C at 3/12 in 2003, C/S for CDP<br>C/S for Fetal distress.<br>Previous episode of Twins, both died. C/S 2008.	2 previous C/S – fill in the problem list. Could've done Sonar to check if 2nd fetus was absorbed.	Clinic	N/A

Supplement to: Kinney MV, George AS, Rhoda N, Pattinson RC, Bergh AM. From pre-implementation to institutionalization: lessons from sustaining a perinatal audit program in South Africa. *Glob Health Sci Pract*. 2023;11(1):e2200213. https://doi.org/10.9745/GHSP-D-22-00213

Table S6.2: Results of applying implementation scoring tool by sub-district

Implementation construct	Progress marker	Instrument item	Case A	Case B	Case C	Case D	Case E
**1. Creating awareness** *(2 points maximum)*	Number and type of (senior) managers involved in implementation process (in relation to size of facility)	Special persons who take specific effort in promoting death reviews including management, professionals, driving forces (contact person, meeting coordinator, other champion)					
		1 point	1	1	1	1	1
		Clear leader(s) involved in establishing and championing death reviews (past or future)					
		1 point	1	1	1	1	1
**2. Adopting the concept** *(2 points maximum)*	Decision to implement MPDSR	Knowledge of the original decision to implement death reviews. If death reviews not yet implemented: has a formal decision been taken?					
		1 point	1	1	1	1	1
	Steering committee	Death review leadership team or steering committee established					
		1 point	1	1	1	1	1
**3. Taking ownership** *(6 points maximum)*	Tools available	Data collection form available					
		1 point	1	1	1	1	1
		Tools include cause of death					
		1 point	1	1	1	1	1
		Tools include modifiable factors					
		1 point	1	1	1	1	1

Implementation construct	Progress marker	Instrument item	Case A	Case B	Case C	Case D	Case E
		Tools include place to follow up on actions taken					
		1 point	1	1	1	1	1
	Meeting process established	Ability to describe or show documentation of meeting process					
		0.5 points	0.5	0.5	0.5	0.5	0.5
		Staff meeting conduct agreement available					
		0.5 points	0	0	0.5	0.5	0.5
	Resources allocated	Allocations from the hospital budget to establish death reviews					
		0.5 points	0	0	0	0	0
		Allocations from other partners to establish death reviews					
		0.5 points	0	0	0	0	0
**4. Evidence of practice** *(7 points maximum)*	Evidence of MPDSR meetings	Meeting minutes available					
		1 point	1	1	1	1	1
		Meeting minutes include action items					
		1 point	1	1	1	1	1
		Meeting minutes include follow up from previous meetings					
		1 point	0	1	1	0	0
		Meeting notes respect confidentiality of staff and patients					
		1 point	1	1	1	1	1
	Orientation for new staff	Face-to-face or written orientation to death reviews					
		1 point	1	0	0.5	0.5	0
	MPDSR data use	Data trends displayed or shared					

Implementation construct	Progress marker	Instrument item	Case A	Case B	Case C	Case D	Case E
		2 points	2	1	2	2	0
**5. Evidence of routine integration** *(7 points maximum)*	Further evidence of practice	Evidence of change based on recommendation arising from death review findings					
		3 points	3	3	3	3	2
	Evidence of routine MPDSR practice	Death review meetings are held at stated interval (e.g. weekly, monthly)					
		1 point	1	1	1	1	1
	Multi-disciplinary meetings	Death review meetings include staff from different disciplines, management					
		2 points	2	2	2	2	2
	Community linkages	Evidence of reporting findings and progress to community					
		1 point	0	0	0	0	0
**6. Evidence of sustainable practice** *(6 points maximum)*	Documented results	Facility records show ongoing death review meetings for at least 1 year					
		2 points	2	2	2	2	2
	Evidence of staff development	Plan in place to ensure all staff receive MPDSR training					
		1 point	1	0	0	0	0
		Evidence that staff have received MPDSR training in the past year					
		1 point	0	0	0	0	0
	Score on the first 5 constructs (divided by 12)	Score on the first 5 constructs will influence sustainability					
		2 points	1.71	1.63	1.79	1.71	1.42
**MAXIMUM TOTAL SCORE**		*30 points*	**25.21**	**23.13**	**25.29**	**24.21**	**20.42**

Supplement to: Kinney MV, George AS, Rhoda N, Pattinson RC, Bergh AM. From pre-implementation to institutionalization: lessons from sustaining a perinatal audit program in South Africa. *Glob Health Sci Pract*. 2023;11(1):e2200213. https://doi.org/10.9745/GHSP-D-22-00213

Box S6.1: Quotes from respondents to support findings

The failure to achieve some progress markers may indicate issue with the pre-determined factors in the tool itself, suggesting these components are either not essential for sustained practice or that the framing of these components need reconsideration.

Budget: Participants indicated no direct budget allocation from the hospital to support the perinatal audit program; however, the time of staff to participate in the related activities was recognized as a related cost but people felt it was worth the expense:

> *But you see that's in our daily business… If I must put a resource price on it [M&M meetings], I mean then it's quite expensive... if you think salary wise. – Hospital manager*

Community: Similarly, we found no evidence of reporting the findings to the community. Three of the case studies indicated functional hospital boards in place with representation from community members, but participants recognized there was no report back specifically about the perinatal audit program. Direct involvement of the affected family in the perinatal audit was perceived as highly sensitive. Participants reported that engagement with the families was managed by the clinical managers and matrons. When asked, participants felt the M&M meetings should be for clinical staff only and should not involve members of the community.

> *They [parents] will be kept up to date but we don't invite them to the M&Ms because we don't want to put them in that spot, but the doctor [clinical manager] will give them feedback… So doctor will communicate with them the whole time. – Quality assurance manager*

REFERENCES

1. Walt G, Shiffman J, Schneider H, et al. 'Doing' Health Policy Analysis: Methodological and Conceptual Reflections and Challenges. Health Policy Plan. 2008;23(5):308-17. doi:10.1093/heapol/czn024
2. Topp SM, Schaaf M, Sriram V, et al. Power Analysis in Health Policy and Systems Research: A Guide to Research Conceptualisation. BMJ Glob Health. 2021;6(11)doi:10.1136/bmjgh-2021-007268
3. Yin RK. Case Study Research Design and Methods (5th Ed.). Thousand Oaks, CA: Sage; 2014.
4. Rhoda NR, Greenfield D, Muller M, et al. Experiences with Perinatal Death Reviews in South Africa--the Perinatal Problem Identification Programme: Scaling up from Programme to Province to Country. BJOG. 2014;121 Suppl 4:160-6. doi:10.1111/1471-0528.12997
5. Belizán M, Bergh AM, Cilliers C, et al. Stages of Change: A Qualitative Study on the Implementation of a Perinatal Audit Programme in South Africa. BMC Health Serv Res. 2011;11:243. doi:10.1186/1472-6963-11-243
6. Kinney MV, Ajayi G, de Graft-Johnson J, et al. "It Might Be a Statistic to Me, but Every Death Matters.": An Assessment of Facility-Level Maternal and Perinatal Death Surveillance and Response

Systems in Four Sub-Saharan African Countries. PLoS One. 2020;15(12):e0243722. doi:10.1371/journal.pone.0243722
7. May C. Towards a General Theory of Implementation. Implement Sci. 2013;8:18. doi:10.1186/1748-5908-8-18
8. Gilson L. Health Policy and Systems Research: A Methodology Reader. Geneva: World Health Organization; 2012.
9. National Department of Health. South African Maternal, Perinatal and Neonatal Health Policy. Pretoria: South African National Department of Health; 2021. Date. Accessed 1 April 2022. https://www.knowledgehub.org.za/system/files/elibdownloads/2021-06/SA%20MPNH%20Policy%2023-6-2021%20signed%20Web%20View%20v2.pdf
10. Woods DL. Improving Neonatal Care in District and Community Health Facilities in South Africa. Paediatr Int Child Health. 2015;35(3):187-91. doi:10.1179/2046905515Y.0000000031
11. NaPeMMCo. Saving Babies 2014-2016: Triennial Report on Perinatal Mortality in South Africa Pretoria: National Department of Health; 2016. Accessed 10 March 2022. https://www.westerncape.gov.za/assets/departments/health/napemmco_triennial_report_2014-2016_saving_babies.pdf
12. Bergh AM, Pattinson R, Belizan M, et al. Completing the Audit Cycle for Quality Care in Perinatal, Newborn and Child Health. Pretoria: MRC Research Unit for Maternal and Infant Health Care Strategies, Univeristy of Pretoria; 2011. Date. Accessed 30 March 2022. https://www.up.ac.za/media/shared/717/PPIP/Saving%20Babies%20Reports/completing-the-audit-cycle-for-quality-care-in-perinatal-newborn-and-child-health.zp194940.pdf
13. Woods DL, Pattinson RC, Greenfield D, et al. Saving Mothers and Babies: Assessing and Reducing Mortality Rates in Your Hospital In: Woods DL, ed. *Perinatal Education Programme Online Courses*. Bettercare Electronic Book Works; 2008.
14. NaPeMMCo. National Perinatal Morbidity and Mortality Committee: Draft Triennial Report (2017-2019) Gebhardt GS, Harper K, eds. Unpublished: National Department of Health; 2020.
